# Computer-based tools provide new insight into the key factors that cause physiological disorders of pistachio rootstocks cultured *in vitro*

**DOI:** 10.1038/s41598-019-46155-2

**Published:** 2019-07-05

**Authors:** Esmaeil Nezami-Alanagh, Ghasem-Ali Garoosi, Mariana Landín, Pedro Pablo Gallego

**Affiliations:** 1 Department of Plant Biology and Soil Science, Faculty of Biology, University of Vigo, 36310 Vigo, Spain; 2 Department of Biotechnology, Faculty of Agriculture and Natural Resources, Imam Khomeini International University (IKIU), Qazvin, Iran; 3 Department of Pharmacology, Pharmacy and Pharmaceutical Technology, Faculty of Pharmacy, University of Santiago, 15782 Santiago de Compostela, Spain; 4 Agrifood Research and Transfer Cluster, Campus da Auga (CITACA), University of Vigo, Ourense, Spain

**Keywords:** Agricultural genetics, Computational models

## Abstract

During the *in vitro* culture of plants some physiological disorders caused major problems that have been associated with culture media composition. The objective of this study was to better understand the abnormal physiological response of two pistachio rootstocks to changes in culture media ingredients. On this purpose, two computer-based tools were employed: design of experiment (DOE) and neurofuzzy logic. DOE was employed to generate a five-dimensional IV-design spaces allowing to reduce the number of treatments from 6,250 to 61. The second one, an artificial intelligence (AI) tool, neurofuzzy logic, was used to understand the cause-effect relationships between the factors studied (25) and seven physiological disorders including shoot-tip necrosis (STN), leaf necrosis (LN), leaf color (LC), basal callus (BC) formation, shoot fasciation (SF), hyperhydricity and epinasty, typically described during pistachio *in vitro* culture. Four out of the seven disorders were successfully modeled, being significantly affected by a limited number of factors. STN and BC were significantly affected by the concentration of EDTA^−^. However, while a low concentration of EDTA^−^ reduces the STN, promotes BC. LN and LC were strongly alleviated by high amounts of thiamine-HCl. Undoubtedly, the results demonstrate the importance of recording and using data related to physiological disorders along with growth parameters when developing suitable culture media for plant tissues. The computer-based tools have been useful to: i) well sample experimental design; ii) reduce the final number of treatments and the experimental work; iii) identify the key factors affecting each disorder; iv) get insight about the causes that promote the appearance of physiological disorders. Our findings demonstrate that the recently AI designed POM media, although not optimal, is the most suitable (favouring growth and limiting physiological abnormalities) media for *in vitro* culture of pistachio compared to those media, currently used.

## Introduction

During the *in vitro* culture of plants is frequent that physiological disorders appear such as shoot-tip necrosis (STN), callus formation at the base of shoots (BC), hyperhydricity, shoot fasciation (SF), epinasty, leaf necrosis (LN) or leaf color (LC), which reduce the yield and the quality of the production.

The necrosis of shoot-tips (STN) was first illustrated by McCown and Sellmer^1^ as a progressive discoloration of the apical meristems that lead the death (necrosis) of the shoot-tip. Among the causes of STN, the growth media type, the cytokinin or the micro-environment of culture vessels have been well-documented^2–5^.

Leaf necrosis (LN) has been characterized by dark or progressively discolored spots. Leaf edge necrosis is frequently reported during *in vitro* culture of variety of species^6–8^. Reed and co-workers^9^ indicated that imbalanced mineral nutrition of commonly utilized culture media such as MS^10^ was associated to the appearance of the disorder in diverse pear germplasm, giving promising improvements on reducing the disorder by increasing CaCl_2_.2H_2_O, KH_2_PO_4_ and MgSO_4_.7H_2_O up to certain concentrations.

The lack of certain nutrients in the culture media also lead variations in leaf color (LC) from green to red in micropropagated shoots of different species^6–9,11^.

The formation of basal callus (BC) is particularly important in the commercial shoot micro-propagation of diverse species, since its appearance probably slows down or even inhibits the absorption of nutrients by the shoots, especially alongside the callus senesces^12^. In melon cultivars, BC has been attributed to the accumulation of calcium in that area of the plant, which can lead to deficiencies of Ca^2+^ in the upper parts of the shoots^13^.

Hyperhydricity has been associated to hypolignification and poor cell wall development^14,15^. Hyperhydric shoots become translucent and water soaked. Leaves become brittle, shiny, dark green and glassy^16^. Moreover, the malformed plantlets do not survive when they are transferred to soil^17^. The type of culture medium or the gelling agent, the mineral nutrients, the plant growth regulators (PGRs), the micro-environment conditions or the containers have been pointed out as factors leading to hyperhydricity in different plant species^15,18–22^. Particularly, in *Pistacia* cultures it has been associated to the type of growth medium^23^ and the cytokinins type and/or concentrations^24–26^.

Shoot fasciation (SF), also named as cristation, is a disorder associated with hyperhydricity and characterized by the development of flatted and abnormal apical meristem, suggesting many stems have fused together^15^. Recently, the causes of the disorder in different plant species have been associated to inadequate type and/or concentration cytokinins as well as a reduced amount of total nitrogen of standard MS medium^27^.

Epinasty is a physiological disorder attributed to the accumulation of produced gases e.g. ethylene in air tight vessels^28^ or insufficient content of mineral nutrients of culture media e.g. calcium^7^. Typical macroscopic symptoms appear in reduced leaf expansion together with promoting downward leaves during micropropagation in a range of species such as *Rosa hybrid* and *Musa sp*.^29,30^.

In pistachio, physiological disorders has been described to occur quite frequently during *in vitro* culture^26^. Those abnormalities have been associated to an imbalance of mineral nutrients in the culture media employed: MS^10^, DKW^31^ or WPM^32^. Several solutions such as readjusting components of media e.g. increasing boron or calcium content or using high (up to 4 mg L^−1^) BAP concentrations^12,24,26,33–38^ have been proposed, but a final solution is far from being found.

The study of the causes of the appearance of physiological disorders in plant tissue cultures has not been addressed very efficiently, mainly due to two reasons. Firstly, plant tissue culture combine a large amount of factors (mineral components, PGRs, vitamins, organic compounds and growth culture conditions) which makes difficult to find the key factor/s causing those physiological abnormalities using factorial designs. Recently, the use of computer-based design of experiment (DOE) has permitted researchers to simultaneously study the effects of multiple factors on a process, with the advantage of a considerable reduction in the number of treatments to carry out^39^. In addition, it allows to obtain general conclusions compared to traditional approaches such as one-factor-at-a-time (OFAT)^40^. Using this methodology, Reed and coworkers^41^ have pointed out the crucial influence of unbalances mineral nutrients on physiological disorders for diverse genotypes of pear. They pointed out that a low concentration of salts categorized as nitrogen (NH_4_NO_3_ or KNO_3_) or mesos (CaCl_2_.2H_2_O, MgSO_4_.7H_2_O and KH_2_PO_4_) gives rise to STN. Many other authors have reached similar conclusions using this type of methodology^7,8,11,42,43^.

Secondly, establishing the effect of a large number of mineral nutrients, vitamins and PGRs on the appearance of physiological disorders, would involve modeling an extremely complex database, which would be difficult using traditional statistical methods^44,45^ but can be easily achieved using artificial intelligence tools^46^. In recent years, the combination of artificial neural networks (ANNs) with fuzzy logic, named as neurofuzzy logic, has been presented as a powerful data mining strategy, which allows the modeling of complex databases and the identification of the key factors to improve a specific response^47^. Neurofuzzy logic systems have two strengths: i) they are able to model very complex databases, and ii) the models are presented as a set of ‘IF- THEN’ rules, which allows researchers to understand the analyzed process and make appropriate decisions to implement optimal culture conditions^48^. This tool has been successfully applied to *in vitro* plant tissue culture in order to model germination rates, shoot multiplication rhizogenesis and acclimatization^45,49,50^. As far as we know, only two physiological disorders, caused by *in vitro* plant tissue culture, were included in neurofuzzy logic models until now^51^.

On this basis, the goal of the present study was to establish the cause of the appearance of the most common physiological disorders in *Pistacia* shoots, as consequence of the use of different genotypes and media formulations (mineral composition, vitamins, glycine and PGRs). To that end, we have combined in one very large database the results obtained from two independent experiments, both developed using of computer-based design of experiment (DOE) to simultaneously study the effects of multiple factors on pistachio tissue culture. Later, we have employed neurofuzzy logic, to model the database and find the key factors involved in the appearance of the physiological disorders.

## Results

**Figure 1:**
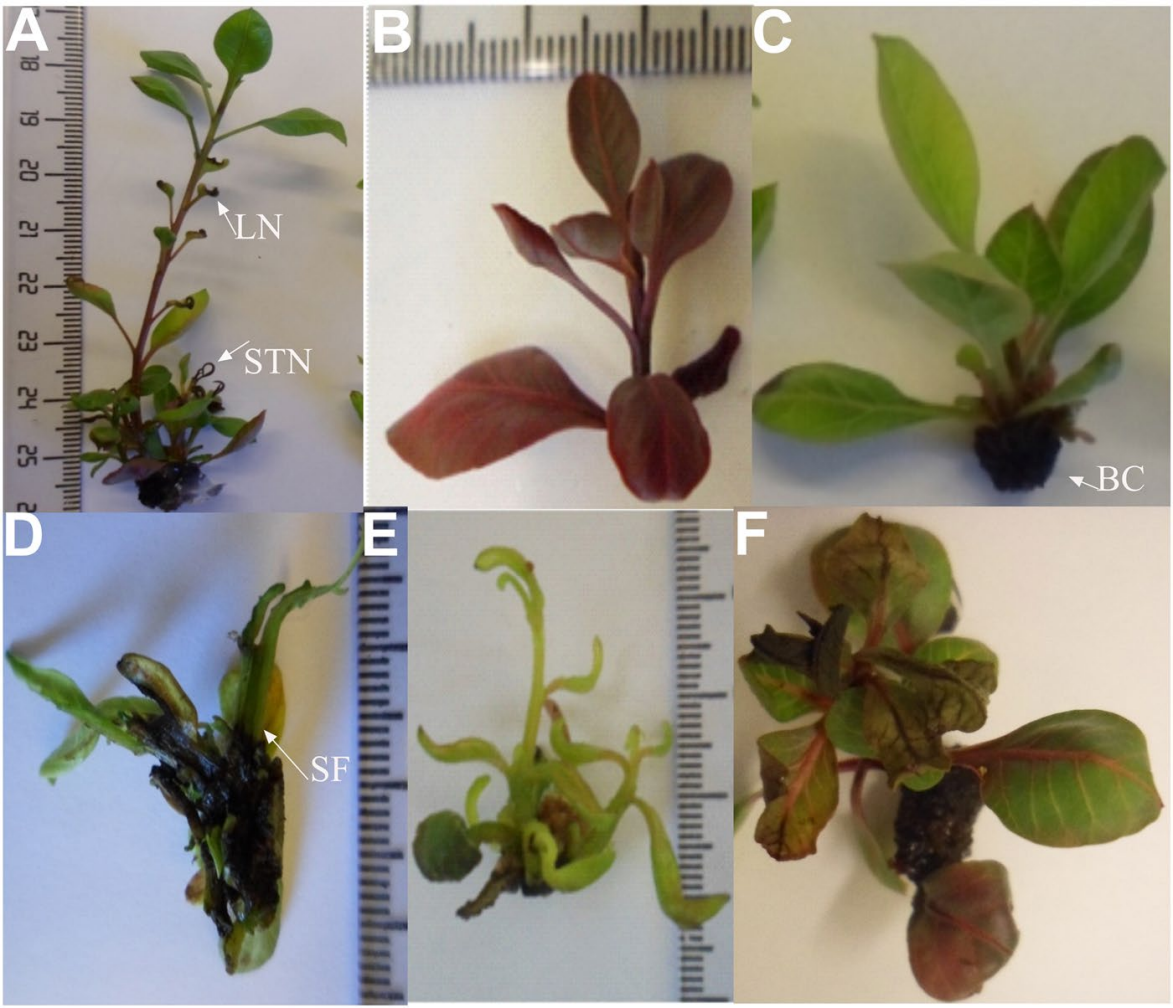
Physiological response of UCB 1 pistachio shoots to compositions of culture media. (**A**) STN and LN; (**B**) LC, (**C**) BC, (**D**) SF, (**E**) hyperhydricity and (**F**) epinasty.

Several physiological disorders were detected after the micropropagation of pistachio as STN & LN (Fig. 1A), LC (Fig. 1B), BC (Fig. 1C), SF (Fig. 1D), hyperhydricity (Fig. 1E) and epinasty (Fig. 1F).

**Figure 2:**
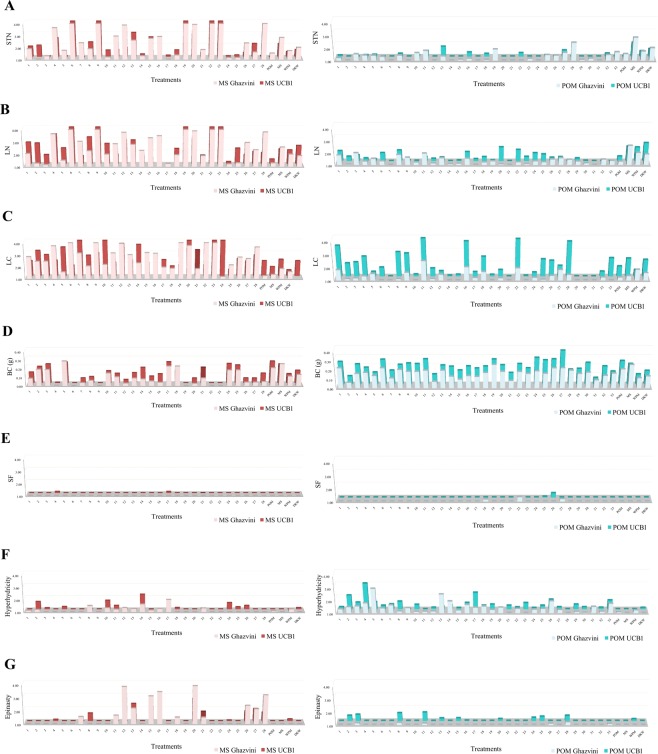
Graphical representation of physiological response of the pistachio shoots to different treatments based on MS and POM together with original mineral nutrients of POM, MS, WPM and DKW; (**A**) STN, (**B**) LN, (**C**) LC, (**D**) BC, (**E**) SF, (**F**) hyperhydricity, and (**G**) epinasty.

Although all data were modeled and analyzed using neurofuzzy logic as described below, we also included here some simple graphs of the results obtained in order to show how difficult is to interpret a cause-effect of factors on the parameters measured using them (Fig. 2). The graphs represent the physiological abnormalities ranged from 1 (no abnormality) to 4 (maximum disorder), except BC which was expressed in grams, being 0 no callus formation. As it can be observed in Fig. 2: (i) all treatments produce physiological abnormalities to some degree, including POM, MS, WPM and DKW media used as controls; (ii) some abnormalities such as STN, LN, LC or BC are frequently (detected in most treatments), while others are rare (only in few treatments) such as SF, hyperhydricity or epinasty; (iii) treatments based on MS media caused higher STN, LN, LC and epinasty than those based in POM media, but contrary, POM based treatments caused higher BC and hyperhydricity compared to MS medium; (iv) some treatments based on MS (6, 9, 19, 22 and 23) caused the maximum disorder (categorized as 4) for STN, LN and LC in both genotypes. On the contrary, those treatments caused the lower BC content; (v) finally, if compared the four basal media used as controls, clearly the pistachio optimized medium (POM) reduced the STN, LN and LC compared to MS, WPM and DKW media. However, the composition of POM or MS promotes more BC than WPM and DKW.

**Figure 3:**
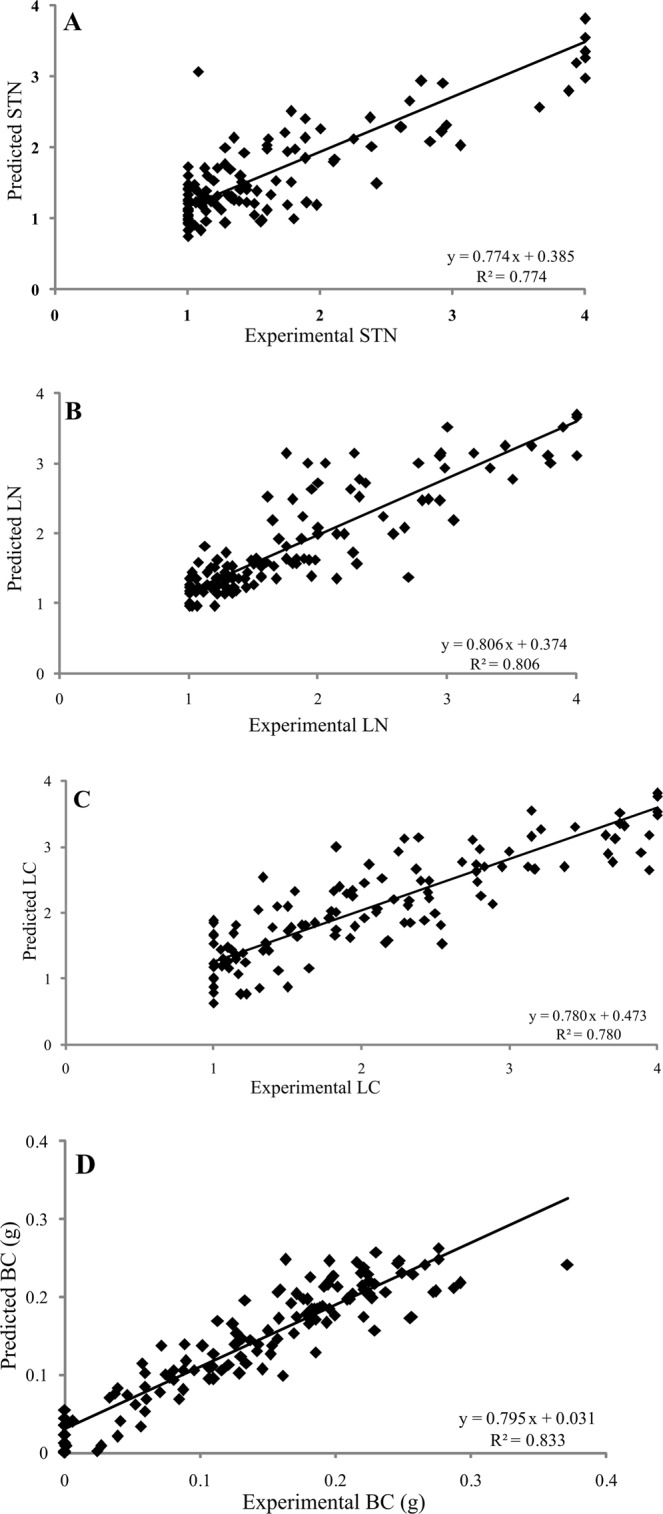
Determination coefficient (*R*^2^) of experimental vs. predicted values achieved by neurofuzzy logic models for the different parameters or *outputs* studied: (**A**) STN, (**B**) LN, (**C**) LC, (**D**) BC.

The previous results showed the great complexity of the micropropagation process and the enormous difficulty for its optimization, since none of the proven treatments was capable of producing healthy shoots without physiological disorders. The establishment of optimal conditions for micropropagation will undoubtedly require the adoption of a compromise solution between the different factors that allow maximizing the growth parameters, simultaneously minimizing the appearance of all abnormalities. In these circumstances, the difficulty in achieving this goal with traditional statistical tools is evident. Nor does the statistics allow us to easily indicate which of the factors determines the appearance of each physiological disorder. The neurofuzzy logic tool was used in order to model the complete data set (Table S1). The results obtained show that this technology has allowed the successful modeling of four of seven analyzed physiological disorders, for which a high determination coefficient (*R*^2^) between the experimental values and those predicted by the model (STN, LN, LC and BC, Fig. 3) was found. On this basis, the components of the studied culture media can be considered as causal agents of the appearance of these four physiological disorders.

The results of STN, LN, BC and LC were explained as a function of independent or interaction of ions, vitamins or PGRs (Table 1), while SF, hyperhydricity and epinasty were not further studied, due to insufficient predictabilities of their models (Train set R^2^ < 70%). Furthermore, the ANOVA *F* ratio for those models was always greater than the *f* critical values together with, assessing the good performance and quality of neurofuzzy logic models.

**Table 1:** Critical factors for each *output* and quality parameters of the neurofuzzy logic models. The *input*s with stronger effect on each *output* have been highlighted.

*Outputs*	Submodel	Significant *inputs*	Train Set R^2^	*F* ratio	df1, df2	*f critical* (α < 0.01)
STN	**1**	**EDTA**^−^ × **K**^+^	77.44	36.82	11, 118	2.40
	2	BAP				
	3	Cl^−^				
	4	Genotype				
	5	Na^+^				
LN	**1**	**Na**^+^ × **Thiamine**-**HCl**	80.63	62.94	8, 121	2.66
	2	Cl^−^				
	3	K^+^				
LC	**1**	**Genotype** × **Thiamine**-**HCl**	78.07	27.05	15, 114	2.19
	2	Fe^2+^ × Mn^2+^				
	3	Cl^−^				
	4	Glycine				
	5	K^+^ × SO_4_^2−^				
BC (g)	**1**	**EDTA**^−^ × **Mn**^**2**+^	82.28	49.82	11, 128	2.40
	2	Glycine				
	3	Cl^−^				
	4	Genotype				
	5	NH_4_^+^				
SF	—	—	4.39	2.92	2, 127	4.77
Hyperhydricity	—	—	11.51	8.26	2, 127	4.77
Epinasty	—	—	21.02	6.60	5, 124	3.16

Neurofuzzy logic model also give information about the key factors involved in each abnormality (Table 1). Just 12 out of 25 *inputs* studied (genotype, NH_4_^+^, K^+^, Na^+^, Fe^2+^, Cl^−^, Mn^2+^, EDTA^−^, SO_4_^2−^, thiamine-HCl, glycine and BAP) affected significantly the disorders.

STN can be explained by five submodels: the interaction of EDTA^−^ and K^+^ as submodel 1 (stronger effect), followed by independent influence of BAP, Cl^−^, genotype and Na^+^ as submodels 2 to 5, respectively. While LN can be explained simply by only three *inputs*: the interaction of Na^+^ and thiamine-HCl (stronger effect), and independent influence of Cl^−^ and K^+^; LC depends on a complex action of eight *inputs*: three interactions (genotype and thiamine-HCl (stronger effect), Fe^2+^ and Mn^2+^ and K^+^ and SO_4_^2−^) and two independent *inputs* (Cl^−^ and glycine). Finally, EDTA^−^ also has had an impact on BC in interaction with Mn^2+^ as submodel 1, followed by independent effects of glycine, Cl^−^, genotype and NH_4_^+^ on the disorder as submodels 2 to 5, respectively Table 1).

Table 2 presents the whole set of ‘IF-THEN’ rules generated by the neurofuzzy logic software for the four abnormalities. To better visualize and interpret those ‘IF-THEN’ rules, both Fig. S1 and Table 3 can be used, which show the ranges corresponding to each word for each variable.

**Table 2:** Rules selection generated by neurofuzzy logic showing the best combination of *inputs* to obtain the highest results for each output. The *inputs* with stronger effects on each *output* indicated by the model have been highlighted.

Rules		Genotype	NH_4_^+^	K^+^	Na^+^	Cl^−^	SO_4_^2−^	Mn^2+^	Fe^2+^	EDTA^−^	Thiamine-HCl	Glycine	BAP		STN	LN	LC	BC(g)	Membership Function
1	IF			Low						Low				THEN	Low				1.00
2				High						Low					Low				1.00
3				Low						Mid					Low				1.00
**4**				**High**						**Mid**					**Low**				**1**.**00**
5				Low						High					High				1.00
**6**				**High**						**High**					**High**				**1**.**00**
7													Low		High				1.00
8													High		Low				1.00
9						Low									High				1.00
10						High									Low				1.00
11		Ghazvini													High				0.55
12		UCB1													Low				0.93
13					Low										Low				0.78
14					Mid										High				0.94
15					High										Low				1.00
16	IF				Low						Low			THEN		Low			0.73
**17**					**High**						**Low**					**High**			**1**.**00**
18					Low						High					Low			1.00
**19**					**High**						**High**					**Low**			**1**.**00**
20						Low										High			0.88
21						Mid										Low			1.00
22						High										Low			0.85
23				Low												Low			0.82
24				Mid												Low			1.00
25				High												High			0.68
**26**	IF	**Ghazvini**									**Low**			THEN			**High**		**1**.**00**
27		UCB1									Low						High		1.00
**28**		**Ghazvini**									**High**						**Low**		**1**.**00**
29		UCB1									High						Low		1.00
30								Low	Low								Low		1.00
31								High	Low								High		1.00
32								Low	Mid								Low		1.00
33								High	Mid								Low		1.00
34								Low	High								High		1.00
35								High	High								High		0.99
36						Low											High		1.00
37						Mid											Low		1.00
38						High											Low		1.00
39												Low					High		1.00
40												High					Low		1.00
41				Low			Low										High		0.81
42				Low			High										Low		1.00
43				High			Low										Low		0.64
44				High			High										High		1.00
45	IF							Low		Low				THEN				High	0.79
**46**								**Mid**		**Low**								**High**	**1**.**00**
47								High		Low								High	1.00
48								Low		High								Low	1.00
**49**								**Mid**		**High**								**Low**	**1**.**00**
50								High		High								Low	0.85
51												Low						High	1.00
52												High						Low	1.00
53						Low												Low	1.00
54						Mid												High	1.00
55						High												High	0.64
56		Ghazvini																Low	0.64
57		UCB1																High	0.80
58			Low															Low	0.60
59			High															High	0.76

**Table 3:** Meaning of the levels of each *inputs* after the fuzzyfication process developed by neurofuzzy logic software after modelling graphically represented in Fig. S1.

*Inputs*	Level	Physiological disorders			
		STN	LN	LC	BC (g)
Cl^−^	Low	0.24 < x < 9.10 mM	0.24 < x < 4.67 mM	0.24 < x < 4.67 mM	0.24 < x < 4.67 mM
	Mid	—	4.67 < x < 13.53 mM	4.67 < x < 13.53 mM	4.67 < x < 13.53 mM
	High	9.10 < x < 17.96 mM	13.53 < x < 17.96 mM	13.53 < x < 17.96 mM	13.53 < x < 17.96 mM
K^+^	Low	0.31 < x < 11.44 mM	0.31 < x < 5.88 mM	0.31 < x < 11.44 mM	
	Mid	—	5.88 < x < 17.00 mM	—	
	High	11.44 < x < 22.56 mM	17.00 < x 22.60 mM	11.44 < x < 22.56 mM	
Na^+^	Low	0.20 < x < 0.40 mM	0.20 < x < 0.61 mM		
	Mid	0.40 < x < 0.80 mM	—		
	High	0.80 < x < 1.00 mM	0.61 < x < 1.01 mM		
EDTA^−^	Low	0.06 < x < 0.17 mM			0.06 < x < 0.28 mM
	Mid	0.17 < x < 0.39 mM			—
	High	0.39 < x < 0.50 mM			0.28 < x < 0.50 mM
NH_4_^+^	Low				4.12 < x < 16.42 mM
	High				16.42 < x < 28.71 mM
SO_4_^2−^	Low			0.49 < x < 6.41 mM	
	High			6.41 < x < 12.33 mM	
Mn^2+^	Low			0.01 < x < 0.28 mM	0.01 < x < 0.15 mM
	Mid			—	0.15 < x < 0.42 mM
	High			0.28 < x < 0.56 mM	0.42 < x < 0.56 mM
Fe^2+^	Low			0.06 < x < 0.17 mM	
	Mid			0.17 < x < 0.39 mM	
	High			0.39 < x < 0.50 mM	
Thiamine-HCl	Low		0.10 < x < 2.70 mgL^−1^	0.10 < x < 2.70 mgL^−1^	
	High		2.70 < x < 5.30 mgL^−1^	2.70 < x < 5.30 mgL^−1^	
Glycine	Low			0.25 < x < 1.13 mgL^−1^	0.25 < x < 1.13 mgL^−1^
	High			1.13 < x < 2.00 mgL^−1^	1.13 < x < 2.00 mgL^−1^
BAP	Low	1.10 < x < 1.30 mg L^−1^			
	High	1.30 < x < 1.50 mg L^−1^			

The ‘IF-THEN’ rules for STN model indicate that the appearance of this disorder in the pistachio shoots is strongly associated to High concentrations of EDTA^−^, regardless K^+^ content (rules 5–6). Additionally, the lowest STN values are also achieved on media including High concentration of BAP and Cl^−^ (rules 8 and 10). ‘UCB1’ shoots showed more resistance than ‘Ghazvini’ rootstock with regard to STN (rules 11–12). Finally, the appearance of STN on the pistachio shoots was affected by Na^+^, being the lowest STN when High amount of sodium is added to the media (rule 15).

It is interesting to note that ‘IF-THEN’ rules pinpointed the beneficial effect of a High content of thiamin-HCl independently of Na^+^ concentration to reduce LN disorder. However, in culture media with a Low content of thiamine-HCl, the inclusion of sodium at High concentration strongly promotes LN disorder (rules 18–19). Chloride and potassium ions also impacted LN but with different thresholds, the lowest LN where obtained at Mid-High concentration of Cl^−^ (rules 21–22) and Low-Mid levels of K^+^ (rules 23–24).

The LC variability is explained by eight out of the 25 *inputs*, with dominant influence of interaction genotype and thiamine-HCl as submodel 1 (Table 1). In both genotypes, the inclusion of a high concentration of thiamine-HCl reduced this disorder, achieving green and healthy leaves (rules 26–29, Table 2). A complex interaction between Fe^2+^ and Mn^2+^ from submodel 2 (Table 1) and LC was found: i) if the culture media had Low concentration of Fe^2+^ and Mn^2+^ promotes low LC and ii) also, when using Mid content of Fe^2+^ was used, independently of Mn^2+^ level (Low or High), low LC was achieved, too (rules 30, 32–33; Table 2). Additionally, the inclusion of Mid-High concentration of chloride or High glycine also alleviates LC disorder (rules 37, 38, 40; Table 2). Finally, submodel 5 (Table 1) shows the complex interaction between K^+^ and SO_4_^2−^ on LC, that can be summarized as both ions should be in opposite concentration (e.g. Low K^+^ with High SO_4_^2−^) to produce the lowest LC disorder (rules 42–43; Table 2).

Callus formation (BC) variability is explained by complex interaction of EDTA^−^ and Mn^2+^ (the strongest effect) and the independent role of glycine, chloride, genotype and ammonium (Table 1). High amount of EDTA^−^ in the media (rules 48–50; Table 2) caused always Low BC, especially if Mn^2+^ is at Mid level (stronger effect; rule 49; Table 2). Pistachio formed low BC on media supplemented with High amount glycine (rule 52; Table 2), Low content of chloride and NH_4_^+^ (rules 53 and 58; Table 2) and ‘Ghazvini’ as genotype (rule 56; Table 2).

## Discussion

In a previous study, POM medium was developed through the use of artificial intelligence tools as fuzzy logic, artificial neural networks and genetic algorithms. Despite of this medium was developed on the basis of a poorly sampled design space, its improvement of growth parameters in *P*. *vera* rootstock micropropagation compared to other general media such as MS, DKW and GNH was demonstrated^52^.

In the present study, media based on MS showed higher STN, LN and LC than those based on POM (Fig. 2A–C), but also lower BC formation (Fig. 2D). These results suggest that although POM is an excellent media for pistachio micropropagation can even be improved by including all those physiological disorders in a future modeling and optimization.

The occurrence of physiological disorders during micropropagation of certain species of *Pistacia* has been attributed to several causes as the culture media composition, the PGRs or the culture system^26,34,37,53–55^, but none of the studies focus on determining the causes of those disorders. We have paid attention to the physiological abnormalities that occurred during two independent micropropagation experiments of *Pistacia* carried out in our laboratory, recording and categorizing data related to several disorders to get insight about the causality of their appearance.

The use an IV-optimal design in both experiments, based on the MS and on the POM medium guaranteed a well sampled design space^39,51^. As controls, both studies included WPM and DKW culture media, as they have been described as causing disorders during pistachio micropropagation^23^. The resulting database covers a wide range of concentrations of each culture media ingredient and permits investigating simultaneously the effects of all media minerals, vitamins, PGRs and genotype on the appearance of abnormalities, an objective that, taking into account the complexity of the generated database, can only be addressed through the AI tools.

Neurofuzzy logic has previously been used as a data mining technique that allow to model and produce intelligent rules to discover key parameters influencing a biological process^46,56^, facilitating the decision making.

In this work, the use of this tool allows us to establish which are the critical factors for each registered physiological disorder, particularly those with the greatest effect (Table 1) and, through the interpretation of the ‘IF-THEN’ rules (Table 2), to understand how these factors modulate the results. The rules are constructed with words whose meaning for each variable is presented in Table 3. Note that the ranges corresponding to each word of each variable may differ in relation to the categorized physiological disorder. This is because the fuzzification process, necessary for the generation of each model, can differ among the disorders.

EDTA^−^ does not fall into mineral nutrients group, but it is an inseparable part of today culture media ingredients and is commonly preferred to other alternative Fe-chelating agents^57^. Murashige and Skoog^10^ used EDTA^−^ at 0.05–0.5 mM in equimolar concentration with iron (Na-Fe-EDTA) to study their effect. An increase in tobacco suspension cultures yield on the media containing up to 0.25 mM of EDTA was found^−^, although they recommended 0.1 mM in the MS media. In agreement with them, pistachio optimized media developed in our laboratory (POM) also includes 0.112 mM of EDTA^52^. The ‘IF-THEN’ rules point out the independent influence of EDTA^−^ on two out of the four disorders (STN and BC). While Low-Mid concentration of EDTA^−^ reduces STN (0.06 <EDTA^−^ < 0.39 mM; Table 3), BC is simultaneously promoted by Low concentrations (0.06 <EDTA^−^< 0.28 mM; Table 3). It is noteworthy to point out that the inclusion of High amount of EDTA^−^ has a negative influence on pistachio proliferation rate in complex interaction with potassium and sulfate^51^. Therefore, it would be postulated that an excess of EDTA^−^ increases STN and decreases of BC by the inhibitory effect on shoot growth that causes in agreement with previous results^51^. Moreover, an excessive level of EDTA^−^ in the culture media is toxic for some plants or chelates other metals, leading to certain mineral deficiency in shoots during their *in vitro* multiplication^57^. Then we recommend using EDTA^−^ at 0.1 mM as suggested in MS and POM.

Potassium can be found in high amounts in plant tissues and has several physiological and biochemical roles. Taiz and Zeiger^58^ pointed out the function of potassium in maintaining turgor and electroneutrality of cells, having also a role as cofactor for more than 40 enzymes involved in cell growth and development. Here, a wide range (0.31–22.56 mM) of potassium was studied. Variations on three physiological disorders (STN, LN and LC) can be explained as a consequence of changes in potassium concentration in the media. Firstly, it causes some significant effect on STN in combination with EDTA^−^, no conclusion can be drawn from the rules obtained. Secondly, Low potassium concentration (0.31 < K^+^ <5 .88 mM; Table 3) reduces the LN disorder. POM media included the lowest concentration of this ion (10.85 mM) in comparison to all control media (ranged from 12.6 in WPM to 20.05 mM in MS), and all media based on POM showed also lower LN (Fig. 2B).

The same comes true in the case of LC, as Low content of potassium together with a high content of SO_4_^2−^ improved LC on the pistachio. Wada and coworkers^7^ using RSM reported the requirement for high concentration of MgSO_4_ to improve LC of diverse pear genotypes. In fact, six out of ten pear genotypes in their study demanded high concentration of MgSO_4_ compared to the MS as control, although ion confounding problem did not allow them to clarify the roles of both ion. Akin and co-workers^59^, using Chi-squared automatic interaction detection data mining algorithm, reported the requirement of a High content of SO_4_^2−^ (8 mM) to increase growth parameters hazelnuts shoots culture, e.g. number of shoots. In agreement with those findings, among the disorders recorded here, LC is affected by SO_4_^2−^, preventing the disorder when a High concentration (6.41 < SO_4_^2−^ < 12.33 mM) of this ion combined with a Low amount of potassium (0.31 < K^+^ < 11.44 mM; Table 3) is used. POM presents a high concentration of SO_4_^2−^ (4.075 mM) compared to MS (1.732 mM) and causes lower LC disorder.

Manganese and iron are two microelements with impact on two out of the four disorders. The interaction between them affects the appearance of LC. The deficiencies in manganese and iron have commonly been associated to appearance of leaf chlorosis^60–62^. Moreover, manganese interacts with EDTA^−^, having an effect on BC. Noticeably, the results indicated that the use of unbalanced amounts of those ions should promote the disorders (rules 30–35 and 45–50; Table 2). By contrast, the inclusion of Fe/EDTA and MnSO_4_, each separately, at a range of 0–1 mM, influenced differently growth parameters of *Gerbera hybrid* cultures, giving 0.1 mM (MS) of the Fe/EDTA as optimum point for the studied parameters^63^.

The vitamins-mixtures of original MS^10^, Gamborg B5^64^ or DKW^31^ are commonly added to the components of culture media used for micropropagation of *Pistacia* species^26,35,37,54,55,65^. The use of computer-based neurofuzzy logic has revealed a linear positive impact of pyridoxine-HCl and nicotinic-acid on promoting some shoot multiplication parameters of pistachio^52^, but, as far as we know, there is no demonstrative conclusion about the influence of vitamin on the physiological disorders occurred during *in vitro* culture of other woody species. The ‘IF-THEN’ rules in the present study showed that the thiamin-HCl and glycine impacted differently on three out of the four disorders. Interestingly, the inclusion of High thiamin-HCl (2.70 < Thiamine-HCl < 5.30 mg L^−1^; Table 3) alleviated strongly the appearance of LN and LC (rules 16–19, 26–29; Table 2). Similarly, the High content of glycine (1.13 < Glycine < 2.00 mg L^−1^; Table 3) reduced strongly LC and BC (rules 39–40, 51–52; Table 2). POM media has a combination of components which clearly reduced LN and LC including high concentration of thiamin (5.3 mM) compared to 0.1 mM in MS, but very low glycine (0.25 mM) compared to MS, WPM or DKW (2 mM). Then for future POM improvement higher amounts of glycine should be tested in order to reduce BC and LN, especially if UCB1 rootstock is used.

The beneficial effect of sodium, as functional nutrient in plants, on increasing growth and alleviating visual symptoms such as leaf chlorosis and necrosis has been previously documented^57,66^. However, there is little information related to the response of *in vitro* plant growth to changes in the sodium concentration of culture media. The use of neurofuzzy logic revealed that sodium has a key role in explaining the variations of two out of the four disorders (STN, and LN), being a single effect for STN parameter or in interaction with thiamine-HCl for LN. Neurofuzzy logic suggests that healthier shoots were obtained when culture medium includes High concentration of sodium. However, treatments with Na^+^ up to Mid concentration (0.20 < Na^+^ < 0.8 mM) promotes shoots length of the pistachio cultures, but at higher concentration caused a negative impact on this parameter (Data not shown). This could explain why Na^+^ at 0.4 mM was selected by a previous model as the optimal for pistachio *in vitro* multiplication^52^.

Genotype is a factor impacting three out of the four disorders in the pistachio cultures. In agreement with our previous report^51^, the cultures of UCB1 showed the lowest and highest frequencies of STN and BC, respectively (rules 11–12, 56–57; Table 2). Also, other studies described that the frequency and type of physiological disorders mediated by unbalanced mineral nutrition varied among genotypes^41^.

McCown and Sellmer^1^ have reported the toxic effects of high chloride concentration (30 mM) on different woody species cultured *in vitro*, when they tried to solve STN problems re-adjusting Ca^2+^ content of MS medium.

Chloride is an ion highly transported in plants with two principal functions: cell division in leaves and shoots, and maintenance of electrical neutrality since it balances the rapid changes that occur in the level of free cations (e.g. K^+^, Mg^2+^ and Na^+^)^57,62^. Mid-High chloride concentration (>4.67 mM; Table 3) should be used to reduce STN, LN and LC (leaf ‘bronzing’) disorders (rules 9–10, 20–22 and 37–38; Table 2). On the contrary, Low concentration (<4.67 mM) is recommended to reduce BC abnormality (rules 53, Table 2). The average concentration of chloride in plant culture media is around 3 mM^57^, however a double amount (6 mM) is used in MS medium. The use of low amounts of chloride in POM, WPM and DKW, 0.47; 1.3 and 2.0 mM, respectively helps to explain the reduction in STN, LN and LC developed in those media, together with the increase in BC. Interesting, although interaction between Cl^−^ and other media components, such as K^+^ or SO_4_^2−^, has been pointed out having an effect of growth parameters^51^, no interaction of chloride with other ions have not been detected here (Table 1).

A range of disorders have also been reported as a result of using insufficient PGRs composition of the culture media^12,23–26,35,36,54,67,68^. BAP not only has a role in promoting growth parameters of pistachio micropropagation^52^, but prevents the appearance of some disorders. In agreement with previous literature^25,69^, the addition of sufficient amount of BAP (1.30 < BAP < 1.50 mg L^−1^; Table 3) strongly reduces the appearance of STN (rules 6–7; Table 2). However, here BAP was conducted just at two fixed concentrations (1.1 and 1.5 mg L^−1^) and new experiments should be carried out in order to find the best combination of PGRs for new optimized culture media for pistachio, combining both growth and disorder parameters in the same database.

Wada and coworkers^6^ asserted the importance of optimizing the nitrogen components content in culture medium to promote multiple elongated shoots and less BC, in diverse pear species. Here, the rules suggest the inclusion of NH_4_^+^ at low content (4.12 < NH_4_^+^ < 16.42 mM; Table 3) to avoid BC in the pistachio shoots. In fact the WPM media with the lowest NH_4_^+^ concentration (5 mM), also produced the lower BC among the control media (MS, POM and DKW). Then, as above mentioned, the final optimization medium for pistachio should include results of several growth parameters along with several physiological disorders to guarantee formulating a robust optimum medium.

Finally, our results demonstrated that some physiological disorders such as SF, hyperhydricity and epinasty were seen infrequently in pistachio and not dependent of the media used. In other species^37,41^, these disorders have been correlated with deficiency of a wide range of mineral nutrients or insufficient composition of PGRs of culture media, but in the range of our study, those disorders could not be properly modeled.

## Conclusions

This study demonstrates that to formulate optimal plant culture medium, the results of both growth parameters and physiological disorders should be considered simultaneously. This fact adds complexity to the design of culture media, increasing the number of treatments to be assayed in proportion to the number of factors and parameters to be taken into account. In this sense computer-based tools such as DOE and AI, have proven to be useful by i) reducing the time and the cost of each experiment (low number of treatments to be tested, only 61 instead of 6,250) but ensuring a well-sampled space design; ii) identifying the key factors affecting each disorder; iii) getting insight about the causes that promote the appearance of physiological disorders in pistachio cultures, and iii) demonstrating that this methodology open a new scenario to design suitable plant tissue culture media. In fact, an AI-designed media for pistachio, POM, was able not only to promote growth parameters but, simultaneously, to reduce physiological disorders, compared with the most used media currently used for pistachio *in vitro* culture.

## Methods

### Plant materials and *in vitro* culture conditions

Shoots of two pistachio rootstocks ‘UCB1’ and *P*. *vera* cv. ‘Ghazvini’ were micro-propagated on MS^10^ and POM^52^ media supplemented with the vitamin-mixture and PGRs composition described in Table S2. Sucrose (30 g L^−1^) and agar (5.7 g L^−1^) were added to each medium. The pH was adjusted to 5.7 prior autoclaving (121 °C, 1 kg cm^−2^ s^−1^ for 20 min). The cultures were kept under 16-h photoperiod (white fluorescent tubes; irradiance of 65 µmol m^−2^ s^−1^) and day/night temperature of 25/20 ± 2 °C and subcultured into a fresh medium every 30 days.

Pistachio explants of approximately 1 cm in length with 1–2 axillary buds were randomly selected before placing them in glass boxes (180 ml) containing 25 ml of the culture medium. After three successive subcultures (30 days interval) on the same culture media, data on STN, LN, SF, hyperhydricity and epinasty were recorded. Responses for these five physiological disorders were rated as 1 = none, 2 = very low, 3 = moderate, 4 = high. For leaf color disorder (LC) response: 1 = green, 2 = pale green, 3 = pink-edged, 4 = bronze-like or brown was recorded. The BCs were weighed and the results expressed in grams.

Each treatment consisted of two replicates glass boxes (180 ml) sealed with caps, containing five explants each. The experiments were carried out in triplicate.

### Design of experiment and data acquisition

The mineral nutrients of MS and POM were subdivided into independent five component factors including (i) KNO_3_ (ii) NH_4_NO_3_, (iii) mesos, (iv) micros, and (v) iron over a range of concentrations expressed in relation to MS medium or POM by attributing 1 × for each as the standard ingredient (Table 4).

**Table 4:** Five-factor mineral nutrients used to construct the experimental design space and concentration range expressed as × levels.

Factors	Mineral nutrients	×MS	×POM
1	KNO_3_	0.0–1.0×	0.5–1.5×
2	NH_4_NO_3_	0.2–1.1×	0.5–1.5×
3 (Mesos)	^*^Ca(NO_3_)_2_.4H_2_O CaCl_2_.2H_2_O KH_2_PO_4_ MgSO4.7H_2_O ^*^K_2_SO_4_ ^*^NaH_2_PO_4_.H_2_O	0.25–3.0×	0.5–1.5×
4 (Micros)	MnSO_4_.4H_2_O ZnSO_4_.7H_2_O CuSO_4_.5H_2_O KI CoCl_2_.6H_2_O H_3_BO_3_ Na_2_MoO_4_.2H_2_O	0.1–4.0×	0.5–3.0×
5 (Iron)	FeSO_4_.7H_2_O Na_2_EDTA.2H_2_O	1.0–5.0×	0.5–3.0×

*These components have been used only in × POM medium.

For MS experiment, the initial five-factor experimental design was a 23-model-point IV-optimal response surface which was sufficient for modeling a quadratic polynomial^51^. The designs were augmented to contain five additional points (in total 28 treatments) to detect additional signal (e.g., curvature) possibly not captured in the design as described by Niedz and Evens^40^ using software Design-Expert^®^8^70^. However for POM experiment, the initial five-factor design was a 23-model-point IV-optimal augmented in 10 additional points (in total 33 treatments). Another four extra points including well-known basal media: MS, POM, WPM and DKW with their original media composition were used as controls. In total, the database included 65-treatment per rootstock and a total of 130 treatments were assayed (Table 5).

**Table 5:** Five-factor IV-design for MS^51^ and POM media together with components of original MS, DKW, WPM and POM as controls (65 treatments).

	Treatments	Factor 1 KNO_3_	Factor 2 NH_4_NO_3_	Factor 3 Mesos	Factor 4 Micros	Factor 5 Iron
×MS medium	#1	1.00	0.20	0.25	4.00	2.33
	#2	0.00	0.20	2.08	4.00	1.00
	#3	1.00	1.10	2.08	0.10	1.00
	#4	0.00	1.10	0.25	1.40	3.67
	#5	0.00	1.10	3.00	4.00	1.00
	#6	0.50	0.20	3.00	0.10	5.00
	#7	0.00	0.20	0.25	0.10	1.00
	#8	0.00	1.10	3.00	4.00	5.00
	#9	0.33	0.50	0.25	0.10	5.00
	#10	0.00	0.50	3.00	0.10	2.33
	#11	1.00	1.10	0.25	4.00	1.00
	#12	0.00	0.20	1.17	2.70	5.00
	#13	1.00	0.65	0.25	0.10	1.00
	#14	0.33	0.20	3.00	4.00	3.67
	#15	1.00	0.20	1.17	0.10	3.67
	#16	0.67	1.10	3.00	0.10	3.67
	#17	0.00	1.10	1.63	0.10	1.00
	#18	1.00	0.80	3.00	4.00	2.33
	#19	1.00	0.80	0.25	2.70	5.00
	#20	1.00	1.10	0.25	0.10	3.67
	#21	0.50	0.65	1.63	2.05	3.00
	#22	1.00	1.10	3.00	2.70	5.00
	#23	1.00	0.20	3.00	2.70	5.00
	#24	0.50	0.65	1.63	2.05	2.00
	#25	0.67	0.20	3.00	1.40	1.00
	#26	0.00	0.80	3.00	1.40	5.00
	#27	0.00	0.80	0.25	4.00	2.33
	#28	0.33	1.10	1.17	4.00	5.00
×POM medium	#1	1.50	1.50	1.38	2.73	0.81
	#2	1.30	0.50	1.50	0.50	3.00
	#3	0.50	1.50	0.50	0.50	0.50
	#4	1.50	0.94	0.50	3.00	3.00
	#5	0.62	1.38	0.50	0.81	2.69
	#6	0.50	0.50	0.93	3.00	1.48
	#7	0.50	0.63	1.45	0.62	2.88
	#8	1.50	1.50	1.50	0.50	0.50
	#9	0.86	0.50	0.50	1.72	0.50
	#10	0.50	1.50	1.50	3.00	3.00
	#11	0.75	0.75	1.50	2.47	0.50
	#12	0.50	0.50	0.50	0.50	3.00
	#13	1.50	1.50	0.50	0.50	1.41
	#14	0.50	0.57	1.50	3.00	3.00
	#15	1.50	0.50	0.97	1.84	3.00
	#16	1.50	0.50	1.50	3.00	0.50
	#17	0.50	1.50	0.50	3.00	3.00
	#18	0.50	0.50	1.50	0.50	0.50
	#19	0.75	1.25	1.20	2.05	1.95
	#20	1.17	0.50	0.50	3.00	1.80
	#21	1.38	1.47	1.38	0.70	3.00
	#22	0.50	0.90	0.50	3.00	0.50
	#23	0.84	0.58	0.94	1.36	2.03
	#24	1.50	0.50	0.50	0.50	0.50
	#25	0.63	1.38	1.38	0.50	0.50
	#26	1.19	1.21	0.93	1.13	0.50
	#27	1.50	1.50	0.50	3.00	0.50
	#28	0.50	1.50	1.50	3.00	0.50
	#29	1.50	1.50	1.50	3.00	3.00
	#30	1.50	0.85	1.50	1.58	1.76
	#31	0.50	1.50	1.50	0.50	3.00
	#32	0.50	0.50	0.50	3.00	3.00
	#33	1.50	1.50	0.50	0.50	3.00
Control	MS	1.00	1.00	1.00	1.00	1.00
	POM	1.00	1.00	1.00	1.00	1.00
	WPM	1.00	1.00	1.00	1.00	1.00
	DKW	1.00	1.00	1.00	1.00	1.00

### Modeling tools

A commercial neurofuzzy logic software, FormRules^®^ 4.03 (Intelligensys, Ltd, UK), was used to model the database generated including 65 treatments per rootstock. The original media macro- and micro-nutrients expressed as salts were converted to their corresponding ions, to avoid ion confounding problems described by Niedz and Evens^40,71^, and merged all in unique database just before modeling. Then, twenty five variables: eighteen calculated ion concentrations (NH_4_^+^, NO_3_^−^, K^+^, Ca^2+^, Mg^2+^, PO_4_^2−^, SO_4_^2−^, Cl^−^, Fe^2+^, BO_3_^−^, Mn^2+^, Zn^2+^, Cu^2+^, MoO_4_^2−^, Na^+^, Co^2+^, I^−^, EDTA^−^), two genotypes (‘Ghazvini’ and ‘UCB1’ rootstocks); four organic compounds: three vitamins (thiamine-HCl, nicotinic-acid and pyridoxine-HCl); glycine and two PGRs (IBA and BA) were included as *inputs*, and seven physiological disorder responses (STN, LN, LC, BC, SF, hyperhydricity, and epinasty) were selected as *outputs* (Table S1). The results for STN and BC obtained in MS media correspond to those described elsewhere^51^ and were included in the dataset (Tables S1 & S2) as controls to compare the effect of the MS versus POM on the physiological disorders.

Modeling was conducted according to our previous methodology^52^ using the training parameters presented in Table 6. Among statistical fitness criteria available in the software, Structural Risk Minimization (SRM) was selected as it is able to find the best model with the minimum generalization error^72^.

**Table 6:** The training parameters setting with neurofuzzy logic.

Critical factors for neurofuzzy logic model
*Minimization parameters*
Ridge regression factor:1e^−6^
*Model selection criteria*
Structural risk minimization (SRM)
C1 = 0.8–0.916; C2 = 4.8
Number of set densities: 2
Set densities: 2, 3
Adapt *nodes*: True
Max.*Inputs* per SubModel: 4
Max. *nodes* per input: 15

Adaptive-spline-modeling-of-data (ASMOD) used by FormRules^®^ enables the models to be split into submodels. The fuzzification processes allows the *input* values to be express by a word (Low, Medium or High) together with a membership degree between 0 and 1^48^. Figure S2 is presented to facilitate the understanding of the linguistic expressions of the variables (Low, Medium and High) obtained by the neurofuzzy logic model^46^.

Independent predictive models were obtained for each physiological disorder, the quality of which was evaluated using the coefficient of determination of the training set (Train Set R^2^) expressed in percentage (for model predictability) and the analysis of variance (ANOVA) parameters (for model accuracy).

Train Set R^2^ values are calculated by following equation^48^.

$${R}^{2}=(1-\frac{{\sum _{i=1}^{n}({y}_{i}-{y^{\prime} }_{i})}^{2}}{\sum _{i=1}^{n}{({y}_{i}-{y^{\prime\prime} }_{i})}^{2}})\times 100 \% $$

where *y*_*i*_ is the experimental point in the data set, *y*_*i*_′ is the predicted point calculated by the model and *y*_*i*_″ is the mean of the dependent variable. Train set R^2^ values between 70 and 99.9% are indicative of acceptable predictabilities, although if R^2^ is higher than 99.9%, the model can be overfitted and the model should be readjusted as described in Colbourn and Rowe^73^. To assess model accuracy, the software uses ANOVA to evaluate statistical differences between predicted and experimental data^52,73^.

## Supplementary information

Supplementary Tables and Figure
