# Supplementary material for: Computer-based tools provide new insight into the key factors that cause physiological disorders of pistachio rootstocks cultured in vitro
Source: Sci Rep. 2019 Jul 5;9:9740. doi: 10.1038/s41598-019-46155-2 (PMC6611901; doi:10.1038/s41598-019-46155-2)

Suplementary tables and figures corresponding to the manuscript:

**Computer-based tools provide new insight into the key factors that cause physiological disorders of pistachio rootstocks cultured *in vitro***

by

Esmaeil Nezami-Alanagh^a,b^, Ghasem-Ali Garoosi^b^, Mariana Landín^c^, Pedro Pablo Gallego^a,d*^

**TABLE S1|** Ion compositions of the different culture media based on the five-factor design space, vitamin-mixture and PGRs to alleviate physiological disorders of pistachio rootstocks. Data for STN and BC for MS medium come from previous dataset^51^.

|  | Treatment N0. | Genotype | Ions  (mM) | | | | | | | | | | | | | | | | | | Vitamins  (mg L^-1^) | | | | PGRs  (mg L^-1^) | | Physiological disorders | | | | | | |
| --- | --- | --- | --- | --- | --- | --- | --- | --- | --- | --- | --- | --- | --- | --- | --- | --- | --- | --- | --- | --- | --- | --- | --- | --- | --- | --- | --- | --- | --- | --- | --- | --- | --- |
|  |  |  | NH_4_^+^ | NO_3_^-^ | K^+^ | Ca^2+^ | Mg^2+^ | PO_4_^2-^ | SO_4_^2-^ | Cl^-^ | Fe^2+^ | EDTA^-^ | BO_3_^-^ | Mn^2+^ | Zn^2+^ | Cu^2+^ | MoO_4_^2-^ | Na^+^ | Co^2+^ | I^-^ | Thiamine- HCl | Nicotinic Acid | Pyridoxine-HCl | Glycine |  | BAP | STN | LN |  | BC (g) | SF | Hyperhydricity | Epinasty |
|  |  |  |  |  |  |  |  |  |  |  |  |  |  |  |  |  |  |  |  |  |  |  |  |  | IBA |  |  |  | LC |  |  |  |  |
| **Control** | **MS** | **UCB1** | **20.61** | **39.41** | **20.05** | **2.99** | **1.50** | **1.25** | **1.73** | **5.99** | **0.100** | **0.100** | **0.100** | **0.100** | **0.030** | **0.000100** | **0.001** | **0.202** | **0.000105** | **0.0050** | **0.10** | **0.50** | **0.50** | **2.00** | **0.10** | **1.10** | **2.10** | **2.20** | **2.40** | **0.23** | **1.00** | **1.00** | **1.00** |
|  | **WPM** | **UCB1** | **5.00** | **9.71** | **12.61** | **3.01** | **1.50** | **1.25** | **7.45** | **1.31** | **0.100** | **0.100** | **0.100** | **0.132** | **0.030** | **0.001001** | **0.001** | **0.202** | **0.000000** | **0.0000** | **1.00** | **0.50** | **0.50** | **2.00** | **0.10** | **1.10** | **1.43** | **2.14** | **1.50** | **0.11** | **1.00** | **1.00** | **1.14** |
|  | **DKW** | **UCB1** | **17.70** | **34.30** | **19.58** | **9.30** | **3.00** | **1.68** | **12.33** | **2.00** | **0.120** | **0.120** | **0.078** | **0.200** | **0.057** | **0.001001** | **0.002** | **0.243** | **0.000000** | **0.0000** | **2.00** | **1.00** | **0.00** | **2.00** | **0.10** | **1.10** | **1.60** | **2.50** | **2.29** | **0.15** | **1.00** | **1.11** | **1.00** |
|  | **MS** | **Ghazvini** | **20.61** | **39.41** | **20.05** | **2.99** | **1.50** | **1.25** | **1.73** | **5.99** | **0.100** | **0.100** | **0.100** | **0.100** | **0.030** | **0.000100** | **0.001** | **0.202** | **0.000105** | **0.0050** | **0.10** | **0.50** | **0.50** | **2.00** | **0.10** | **1.10** | **2.83** | **2.58** | **1.33** | **0.26** | **1.00** | **1.00** | **1.00** |
|  | **WPM** | **Ghazvini** | **5.00** | **9.71** | **12.61** | **3.01** | **1.50** | **1.25** | **7.45** | **1.31** | **0.100** | **0.100** | **0.100** | **0.132** | **0.030** | **0.001001** | **0.001** | **0.202** | **0.000000** | **0.0000** | **1.00** | **0.50** | **0.50** | **2.00** | **0.10** | **1.10** | **1.73** | **2.00** | **1.83** | **0.11** | **1.00** | **1.22** | **1.00** |
|  | **DKW** | **Ghazvini** | **17.70** | **34.30** | **19.58** | **9.30** | **3.00** | **1.68** | **12.33** | **2.00** | **0.120** | **0.120** | **0.078** | **0.200** | **0.057** | **0.001001** | **0.002** | **0.243** | **0.000000** | **0.0000** | **2.00** | **1.00** | **0.00** | **2.00** | **0.10** | **1.10** | **2.00** | **1.88** | **1.57** | **0.13** | **1.00** | **1.00** | **1.00** |
| **×MS medium** | 1 | UCB1 | 4.12 | 22.92 | 19.13 | 0.75 | 0.38 | 0.31 | 1.13 | 1.50 | 0.234 | 0.234 | 0.401 | 0.400 | 0.120 | 0.000401 | 0.004 | 0.476 | 0.000420 | 0.0200 | 0.10 | 0.50 | 0.50 | 2.00 | 0.10 | 1.10 | 1.89 | 2.78 | 3.17 | 0.13 | 1.00 | 1.00 | 1.00 |
|  | 2 | UCB1 | 4.12 | 4.12 | 2.62 | 6.23 | 3.13 | 2.60 | 3.75 | 12.47 | 0.100 | 0.100 | 0.401 | 0.400 | 0.120 | 0.000401 | 0.004 | 0.209 | 0.000420 | 0.0200 | 0.10 | 0.50 | 0.50 | 2.00 | 0.10 | 1.10 | 1.98 | 2.70 | 2.81 | 0.19 | 1.00 | 1.63 | 1.00 |
|  | 3 | UCB1 | 22.68 | 41.47 | 21.40 | 6.23 | 3.13 | 2.60 | 3.24 | 12.47 | 0.100 | 0.100 | 0.010 | 0.010 | 0.003 | 0.000010 | 0.000 | 0.201 | 0.000011 | 0.0005 | 0.10 | 0.50 | 0.50 | 2.00 | 0.10 | 1.10 | 1.05 | 1.75 | 2.33 | 0.23 | 1.00 | 1.10 | 1.00 |
|  | 4 | UCB1 | 22.68 | 22.68 | 0.32 | 0.75 | 0.38 | 0.31 | 0.93 | 1.50 | 0.368 | 0.367 | 0.140 | 0.140 | 0.042 | 0.000140 | 0.001 | 0.737 | 0.000147 | 0.0070 | 0.10 | 0.50 | 0.50 | 2.00 | 0.10 | 1.10 | 2.61 | 3.44 | 3.44 | 0.01 | 1.11 | 1.00 | 1.11 |
|  | 5 | UCB1 | 22.68 | 22.68 | 3.77 | 8.98 | 4.50 | 3.75 | 5.12 | 17.96 | 0.100 | 0.100 | 0.401 | 0.400 | 0.120 | 0.000401 | 0.004 | 0.209 | 0.000420 | 0.0200 | 0.10 | 0.50 | 0.50 | 2.00 | 0.10 | 1.10 | 1.05 | 2.30 | 1.90 | 0.25 | 1.00 | 1.20 | 1.00 |
|  | 6 | UCB1 | 4.12 | 13.52 | 13.14 | 8.98 | 4.50 | 3.75 | 5.02 | 17.96 | 0.502 | 0.501 | 0.010 | 0.010 | 0.003 | 0.000010 | 0.000 | 1.002 | 0.000011 | 0.0005 | 0.10 | 0.50 | 0.50 | 2.00 | 0.10 | 1.10 | 4.00 | 4.00 | 4.00 | 0.00 | 1.00 | 1.00 | 1.00 |
|  | 7 | UCB1 | 4.12 | 4.12 | 0.31 | 0.75 | 0.38 | 0.31 | 0.49 | 1.50 | 0.100 | 0.100 | 0.010 | 0.010 | 0.003 | 0.000010 | 0.000 | 0.201 | 0.000011 | 0.0005 | 0.10 | 0.50 | 0.50 | 2.00 | 0.10 | 1.10 | 1.00 | 1.65 | 2.75 | 0.06 | 1.00 | 1.00 | 1.00 |
|  | 8 | UCB1 | 22.68 | 22.68 | 3.77 | 8.98 | 4.50 | 3.75 | 5.53 | 17.96 | 0.502 | 0.501 | 0.401 | 0.400 | 0.120 | 0.000401 | 0.004 | 1.010 | 0.000420 | 0.0200 | 0.10 | 0.50 | 0.50 | 2.00 | 0.10 | 1.10 | 2.25 | 3.20 | 2.95 | 0.07 | 1.00 | 1.00 | 1.60 |
|  | 9 | UCB1 | 10.31 | 16.57 | 6.58 | 0.75 | 0.38 | 0.31 | 0.89 | 1.50 | 0.502 | 0.501 | 0.010 | 0.010 | 0.003 | 0.000010 | 0.000 | 1.002 | 0.000011 | 0.0005 | 0.10 | 0.50 | 0.50 | 2.00 | 0.10 | 1.10 | 4.00 | 4.00 | 4.00 | 0.00 | 1.00 | 1.00 | 1.00 |
|  | 10 | UCB1 | 10.31 | 10.31 | 3.75 | 8.98 | 4.50 | 3.75 | 4.75 | 17.96 | 0.234 | 0.234 | 0.010 | 0.010 | 0.003 | 0.000010 | 0.000 | 0.468 | 0.000011 | 0.0005 | 0.10 | 0.50 | 0.50 | 2.00 | 0.10 | 1.10 | 1.56 | 2.67 | 2.17 | 0.14 | 1.00 | 1.72 | 1.00 |
|  | 11 | UCB1 | 22.68 | 41.47 | 19.13 | 0.75 | 0.38 | 0.31 | 1.00 | 1.50 | 0.100 | 0.100 | 0.401 | 0.400 | 0.120 | 0.000401 | 0.004 | 0.209 | 0.000420 | 0.0200 | 0.10 | 0.50 | 0.50 | 2.00 | 0.10 | 1.10 | 1.60 | 1.80 | 3.75 | 0.11 | 1.00 | 1.30 | 1.00 |
|  | 12 | UCB1 | 4.12 | 4.12 | 1.47 | 3.49 | 1.75 | 1.46 | 2.60 | 6.98 | 0.502 | 0.501 | 0.271 | 0.270 | 0.081 | 0.000270 | 0.003 | 1.008 | 0.000284 | 0.0135 | 0.10 | 0.50 | 0.50 | 2.00 | 0.10 | 1.10 | 1.78 | 2.94 | 2.39 | 0.04 | 1.00 | 1.00 | 1.00 |
|  | 13 | UCB1 | 13.40 | 32.19 | 19.11 | 0.75 | 0.38 | 0.31 | 0.49 | 1.50 | 0.100 | 0.100 | 0.010 | 0.010 | 0.003 | 0.000010 | 0.000 | 0.201 | 0.000011 | 0.0005 | 0.10 | 0.50 | 0.50 | 2.00 | 0.10 | 1.10 | 3.06 | 2.94 | 3.67 | 0.12 | 1.00 | 1.00 | 2.33 |
|  | 14 | UCB1 | 4.12 | 10.39 | 10.03 | 8.98 | 4.50 | 3.75 | 5.39 | 17.96 | 0.368 | 0.367 | 0.401 | 0.400 | 0.120 | 0.000401 | 0.004 | 0.743 | 0.000420 | 0.0200 | 0.10 | 0.50 | 0.50 | 2.00 | 0.10 | 1.10 | 1.22 | 1.61 | 2.11 | 0.18 | 1.00 | 2.22 | 1.00 |
|  | 15 | UCB1 | 4.12 | 22.92 | 20.25 | 3.49 | 1.75 | 1.46 | 2.13 | 6.98 | 0.368 | 0.367 | 0.010 | 0.010 | 0.003 | 0.000010 | 0.000 | 0.735 | 0.000011 | 0.0005 | 0.10 | 0.50 | 0.50 | 2.00 | 0.10 | 1.10 | 2.68 | 2.98 | 2.78 | 0.08 | 1.00 | 1.00 | 2.10 |
|  | 16 | UCB1 | 22.68 | 35.21 | 16.28 | 8.98 | 4.50 | 3.75 | 4.88 | 17.96 | 0.368 | 0.367 | 0.010 | 0.010 | 0.003 | 0.000010 | 0.000 | 0.735 | 0.000011 | 0.0005 | 0.10 | 0.50 | 0.50 | 2.00 | 0.10 | 1.10 | 1.75 | 2.31 | 2.38 | 0.11 | 1.00 | 1.00 | 1.69 |
|  | 17 | UCB1 | 22.68 | 22.68 | 2.03 | 4.86 | 2.44 | 2.03 | 2.55 | 9.73 | 0.100 | 0.100 | 0.010 | 0.010 | 0.003 | 0.000010 | 0.000 | 0.201 | 0.000011 | 0.0005 | 0.10 | 0.50 | 0.50 | 2.00 | 0.10 | 1.10 | 1.11 | 1.00 | 1.83 | 0.25 | 1.11 | 1.44 | 1.00 |
|  | 18 | UCB1 | 16.49 | 35.29 | 22.56 | 8.98 | 4.50 | 3.75 | 5.26 | 17.96 | 0.234 | 0.234 | 0.401 | 0.400 | 0.120 | 0.000401 | 0.004 | 0.476 | 0.000420 | 0.0200 | 0.10 | 0.50 | 0.50 | 2.00 | 0.10 | 1.10 | 1.56 | 2.25 | 1.94 | 0.19 | 1.00 | 1.13 | 1.00 |
|  | 19 | UCB1 | 16.49 | 35.29 | 19.12 | 0.75 | 0.38 | 0.31 | 1.23 | 1.50 | 0.502 | 0.501 | 0.271 | 0.270 | 0.081 | 0.000270 | 0.003 | 1.008 | 0.000284 | 0.0135 | 0.10 | 0.50 | 0.50 | 2.00 | 0.10 | 1.10 | 4.00 | 4.00 | 4.00 | 0.00 | 1.00 | 1.00 | 1.00 |
|  | 20 | UCB1 | 22.68 | 41.47 | 19.11 | 0.75 | 0.38 | 0.31 | 0.76 | 1.50 | 0.368 | 0.367 | 0.010 | 0.010 | 0.003 | 0.000010 | 0.000 | 0.735 | 0.000011 | 0.0005 | 0.10 | 0.50 | 0.50 | 2.00 | 0.10 | 1.10 | 2.93 | 3.00 | 3.21 | 0.06 | 1.00 | 1.00 | 2.71 |
|  | 21 | UCB1 | 13.40 | 22.80 | 11.44 | 4.86 | 2.44 | 2.03 | 3.01 | 9.73 | 0.301 | 0.301 | 0.206 | 0.205 | 0.061 | 0.000205 | 0.002 | 0.605 | 0.000215 | 0.0103 | 0.10 | 0.50 | 0.50 | 2.00 | 0.10 | 1.10 | 1.33 | 1.70 | 1.93 | 0.18 | 1.00 | 1.08 | 1.73 |
|  | 22 | UCB1 | 22.68 | 41.47 | 22.55 | 8.98 | 4.50 | 3.75 | 5.36 | 17.96 | 0.502 | 0.501 | 0.271 | 0.270 | 0.081 | 0.000270 | 0.003 | 1.008 | 0.000284 | 0.0135 | 0.10 | 0.50 | 0.50 | 2.00 | 0.10 | 1.10 | 4.00 | 4.00 | 4.00 | 0.00 | 1.00 | 1.00 | 1.00 |
|  | 23 | UCB1 | 4.12 | 22.92 | 22.55 | 8.98 | 4.50 | 3.75 | 5.36 | 17.96 | 0.502 | 0.501 | 0.271 | 0.270 | 0.081 | 0.000270 | 0.003 | 1.008 | 0.000284 | 0.0135 | 0.10 | 0.50 | 0.50 | 2.00 | 0.10 | 1.10 | 4.00 | 4.00 | 4.00 | 0.00 | 1.00 | 1.00 | 1.00 |
|  | 24 | UCB1 | 13.40 | 22.80 | 11.44 | 4.86 | 2.44 | 2.03 | 2.91 | 9.73 | 0.201 | 0.200 | 0.206 | 0.205 | 0.061 | 0.000205 | 0.002 | 0.405 | 0.000215 | 0.0103 | 0.10 | 0.50 | 0.50 | 2.00 | 0.10 | 1.10 | 1.06 | 1.17 | 1.53 | 0.23 | 1.00 | 1.53 | 1.06 |
|  | 25 | UCB1 | 4.12 | 16.65 | 16.28 | 8.98 | 4.50 | 3.75 | 4.79 | 17.96 | 0.100 | 0.100 | 0.140 | 0.140 | 0.042 | 0.000140 | 0.001 | 0.203 | 0.000147 | 0.0070 | 0.10 | 0.50 | 0.50 | 2.00 | 0.10 | 1.10 | 1.14 | 2.27 | 2.45 | 0.21 | 1.00 | 1.18 | 1.00 |
|  | 26 | UCB1 | 16.49 | 16.49 | 3.75 | 8.98 | 4.50 | 3.75 | 5.19 | 17.96 | 0.502 | 0.501 | 0.140 | 0.140 | 0.042 | 0.000140 | 0.001 | 1.005 | 0.000147 | 0.0070 | 0.10 | 0.50 | 0.50 | 2.00 | 0.10 | 1.10 | 1.35 | 1.75 | 2.25 | 0.06 | 1.00 | 1.30 | 1.75 |
|  | 27 | UCB1 | 16.49 | 16.49 | 0.33 | 0.75 | 0.38 | 0.31 | 1.13 | 1.50 | 0.234 | 0.234 | 0.401 | 0.400 | 0.120 | 0.000401 | 0.004 | 0.476 | 0.000420 | 0.0200 | 0.10 | 0.50 | 0.50 | 2.00 | 0.10 | 1.10 | 2.11 | 2.00 | 2.78 | 0.06 | 1.00 | 1.00 | 1.94 |
|  | 28 | UCB1 | 22.68 | 28.94 | 7.74 | 3.49 | 1.75 | 1.46 | 2.77 | 6.98 | 0.502 | 0.501 | 0.401 | 0.400 | 0.120 | 0.000401 | 0.004 | 1.010 | 0.000420 | 0.0200 | 0.10 | 0.50 | 0.50 | 2.00 | 0.10 | 1.10 | 1.08 | 1.92 | 2.29 | 0.12 | 1.00 | 1.00 | 1.67 |
|  | 1 | Ghazvini | 4.12 | 22.92 | 19.13 | 0.75 | 0.38 | 0.31 | 1.13 | 1.50 | 0.234 | 0.234 | 0.401 | 0.400 | 0.120 | 0.000401 | 0.004 | 0.476 | 0.000420 | 0.0200 | 0.10 | 0.50 | 0.50 | 2.00 | 0.10 | 1.10 | 1.89 | 2.06 | 2.83 | 0.08 | 1.00 | 1.11 | 1.00 |
|  | 2 | Ghazvini | 4.12 | 4.12 | 2.62 | 6.23 | 3.13 | 2.60 | 3.75 | 12.47 | 0.100 | 0.100 | 0.401 | 0.400 | 0.120 | 0.000401 | 0.004 | 0.209 | 0.000420 | 0.0200 | 0.10 | 0.50 | 0.50 | 2.00 | 0.10 | 1.10 | 1.00 | 1.26 | 2.45 | 0.19 | 1.00 | 1.18 | 1.00 |
|  | 3 | Ghazvini | 22.68 | 41.47 | 21.40 | 6.23 | 3.13 | 2.60 | 3.24 | 12.47 | 0.100 | 0.100 | 0.010 | 0.010 | 0.003 | 0.000010 | 0.000 | 0.201 | 0.000011 | 0.0005 | 0.10 | 0.50 | 0.50 | 2.00 | 0.10 | 1.10 | 1.28 | 1.13 | 2.46 | 0.19 | 1.00 | 1.00 | 1.00 |
|  | 4 | Ghazvini | 22.68 | 22.68 | 0.32 | 0.75 | 0.38 | 0.31 | 0.93 | 1.50 | 0.368 | 0.367 | 0.140 | 0.140 | 0.042 | 0.000140 | 0.001 | 0.738 | 0.000147 | 0.0070 | 0.10 | 0.50 | 0.50 | 2.00 | 0.10 | 1.10 | 3.65 | 3.65 | 3.75 | 0.00 | 1.00 | 1.00 | 1.00 |
|  | 5 | Ghazvini | 22.68 | 22.68 | 3.77 | 8.98 | 4.50 | 3.75 | 5.12 | 17.96 | 0.100 | 0.100 | 0.401 | 0.400 | 0.120 | 0.000401 | 0.004 | 0.209 | 0.000420 | 0.0200 | 0.10 | 0.50 | 0.50 | 2.00 | 0.10 | 1.10 | 1.75 | 1.50 | 1.55 | 0.29 | 1.00 | 1.00 | 1.00 |
|  | 6 | Ghazvini | 4.12 | 13.52 | 13.14 | 8.98 | 4.50 | 3.75 | 5.02 | 17.96 | 0.502 | 0.501 | 0.010 | 0.010 | 0.003 | 0.000010 | 0.000 | 1.002 | 0.000011 | 0.0005 | 0.10 | 0.50 | 0.50 | 2.00 | 0.10 | 1.10 | 4.00 | 4.00 | 4.00 | 0.00 | 1.00 | 1.00 | 1.00 |
|  | 7 | Ghazvini | 4.12 | 4.12 | 0.31 | 0.75 | 0.38 | 0.31 | 0.49 | 1.50 | 0.100 | 0.100 | 0.010 | 0.010 | 0.003 | 0.000010 | 0.000 | 0.201 | 0.000011 | 0.0005 | 0.10 | 0.50 | 0.50 | 2.00 | 0.10 | 1.10 | 2.39 | 3.05 | 3.15 | 0.03 | 1.00 | 1.00 | 1.55 |
|  | 8 | Ghazvini | 22.68 | 22.68 | 3.77 | 8.98 | 4.50 | 3.75 | 5.53 | 17.96 | 0.502 | 0.501 | 0.401 | 0.400 | 0.120 | 0.000401 | 0.004 | 1.010 | 0.000420 | 0.0200 | 0.10 | 0.50 | 0.50 | 2.00 | 0.10 | 1.10 | 1.89 | 2.28 | 2.06 | 0.06 | 1.00 | 1.50 | 1.00 |
|  | 9 | Ghazvini | 10.31 | 16.57 | 6.58 | 0.75 | 0.38 | 0.31 | 0.89 | 1.50 | 0.502 | 0.501 | 0.010 | 0.010 | 0.003 | 0.000010 | 0.000 | 1.002 | 0.000011 | 0.0005 | 0.10 | 0.50 | 0.50 | 2.00 | 0.10 | 1.10 | 4.00 | 4.00 | 4.00 | 0.00 | 1.00 | 1.00 | 1.00 |
|  | 10 | Ghazvini | 10.31 | 10.31 | 3.75 | 8.98 | 4.50 | 3.75 | 4.75 | 17.96 | 0.234 | 0.234 | 0.010 | 0.010 | 0.003 | 0.000010 | 0.000 | 0.468 | 0.000011 | 0.0005 | 0.10 | 0.50 | 0.50 | 2.00 | 0.10 | 1.10 | 1.18 | 2.00 | 2.18 | 0.15 | 1.00 | 1.32 | 1.00 |
|  | 11 | Ghazvini | 22.68 | 41.47 | 19.13 | 0.75 | 0.38 | 0.31 | 1.00 | 1.50 | 0.100 | 0.100 | 0.401 | 0.400 | 0.120 | 0.000401 | 0.004 | 0.209 | 0.000420 | 0.0200 | 0.10 | 0.50 | 0.50 | 2.00 | 0.10 | 1.10 | 2.95 | 2.85 | 3.15 | 0.10 | 1.00 | 1.20 | 1.65 |
|  | 12 | Ghazvini | 4.12 | 4.12 | 1.47 | 3.49 | 1.75 | 1.46 | 2.60 | 6.98 | 0.502 | 0.501 | 0.271 | 0.270 | 0.081 | 0.000270 | 0.003 | 1.008 | 0.000284 | 0.0135 | 0.10 | 0.50 | 0.50 | 2.00 | 0.10 | 1.10 | 3.88 | 3.78 | 3.94 | 0.03 | 1.00 | 1.33 | 3.83 |
|  | 13 | Ghazvini | 13.40 | 32.19 | 19.11 | 0.75 | 0.38 | 0.31 | 0.49 | 1.50 | 0.100 | 0.100 | 0.010 | 0.010 | 0.003 | 0.000010 | 0.000 | 0.201 | 0.000011 | 0.0005 | 0.10 | 0.50 | 0.50 | 2.00 | 0.10 | 1.10 | 2.61 | 2.80 | 3.00 | 0.09 | 1.00 | 1.25 | 2.20 |
|  | 14 | Ghazvini | 4.12 | 10.39 | 10.03 | 8.98 | 4.50 | 3.75 | 5.39 | 17.96 | 0.368 | 0.367 | 0.401 | 0.400 | 0.120 | 0.000401 | 0.004 | 0.743 | 0.000420 | 0.0200 | 0.10 | 0.50 | 0.50 | 2.00 | 0.10 | 1.10 | 1.29 | 2.31 | 2.31 | 0.08 | 1.00 | 1.63 | 1.00 |
|  | 15 | Ghazvini | 4.12 | 22.92 | 20.25 | 3.49 | 1.75 | 1.46 | 2.13 | 6.98 | 0.368 | 0.367 | 0.010 | 0.010 | 0.003 | 0.000010 | 0.000 | 0.735 | 0.000011 | 0.0005 | 0.10 | 0.50 | 0.50 | 2.00 | 0.10 | 1.10 | 2.76 | 3.33 | 3.18 | 0.05 | 1.00 | 1.13 | 3.12 |
|  | 16 | Ghazvini | 22.68 | 35.21 | 16.28 | 8.98 | 4.50 | 3.75 | 4.88 | 17.96 | 0.368 | 0.367 | 0.010 | 0.010 | 0.003 | 0.000010 | 0.000 | 0.735 | 0.000011 | 0.0005 | 0.10 | 0.50 | 0.50 | 2.00 | 0.10 | 1.10 | 2.92 | 3.50 | 3.13 | 0.04 | 1.00 | 1.25 | 3.44 |
|  | 17 | Ghazvini | 22.68 | 22.68 | 2.03 | 4.86 | 2.44 | 2.03 | 2.55 | 9.73 | 0.100 | 0.100 | 0.010 | 0.010 | 0.003 | 0.000010 | 0.000 | 0.201 | 0.000011 | 0.0005 | 0.10 | 0.50 | 0.50 | 2.00 | 0.10 | 1.10 | 1.00 | 1.23 | 1.95 | 0.23 | 1.00 | 2.00 | 1.00 |
|  | 18 | Ghazvini | 16.49 | 35.29 | 22.56 | 8.98 | 4.50 | 3.75 | 5.26 | 17.96 | 0.234 | 0.234 | 0.401 | 0.400 | 0.120 | 0.000401 | 0.004 | 0.476 | 0.000420 | 0.0200 | 0.10 | 0.50 | 0.50 | 2.00 | 0.10 | 1.10 | 1.35 | 1.95 | 1.85 | 0.23 | 1.00 | 1.00 | 1.50 |
|  | 19 | Ghazvini | 16.49 | 35.29 | 19.12 | 0.75 | 0.38 | 0.31 | 1.23 | 1.50 | 0.502 | 0.501 | 0.271 | 0.270 | 0.081 | 0.000270 | 0.003 | 1.008 | 0.000284 | 0.0135 | 0.10 | 0.50 | 0.50 | 2.00 | 0.10 | 1.10 | 4.00 | 4.00 | 4.00 | 0.00 | 1.00 | 1.00 | 1.00 |
|  | 20 | Ghazvini | 22.68 | 41.47 | 19.11 | 0.75 | 0.38 | 0.31 | 0.76 | 1.50 | 0.368 | 0.367 | 0.010 | 0.010 | 0.003 | 0.000010 | 0.000 | 0.735 | 0.000011 | 0.0005 | 0.10 | 0.50 | 0.50 | 2.00 | 0.10 | 1.10 | 3.93 | 3.89 | 3.78 | 0.02 | 1.00 | 1.00 | 3.89 |
|  | 21 | Ghazvini | 13.40 | 22.80 | 11.44 | 4.86 | 2.44 | 2.03 | 3.01 | 9.73 | 0.301 | 0.301 | 0.206 | 0.205 | 0.061 | 0.000205 | 0.002 | 0.605 | 0.000215 | 0.0103 | 0.10 | 0.50 | 0.50 | 2.00 | 0.10 | 1.10 | 1.81 | 1.88 | 1.83 | 0.09 | 1.00 | 1.30 | 1.50 |
|  | 22 | Ghazvini | 22.68 | 41.47 | 22.55 | 8.98 | 4.50 | 3.75 | 5.36 | 17.96 | 0.502 | 0.501 | 0.271 | 0.270 | 0.081 | 0.000270 | 0.003 | 1.008 | 0.000284 | 0.0135 | 0.10 | 0.50 | 0.50 | 2.00 | 0.10 | 1.10 | 4.00 | 4.00 | 4.00 | 0.00 | 1.00 | 1.00 | 1.00 |
|  | 23 | Ghazvini | 4.12 | 22.92 | 22.55 | 8.98 | 4.50 | 3.75 | 5.36 | 17.96 | 0.502 | 0.501 | 0.271 | 0.270 | 0.081 | 0.000270 | 0.003 | 1.008 | 0.000284 | 0.0135 | 0.10 | 0.50 | 0.50 | 2.00 | 0.10 | 1.10 | 4.00 | 4.00 | 4.00 | 0.00 | 1.00 | 1.00 | 1.00 |
|  | 24 | Ghazvini | 13.40 | 22.80 | 11.44 | 4.86 | 2.44 | 2.03 | 2.91 | 9.73 | 0.201 | 0.200 | 0.206 | 0.205 | 0.061 | 0.000205 | 0.002 | 0.405 | 0.000215 | 0.0103 | 0.10 | 0.50 | 0.50 | 2.00 | 0.10 | 1.10 | 1.13 | 1.20 | 1.15 | 0.18 | 1.00 | 1.00 | 1.00 |
|  | 25 | Ghazvini | 4.12 | 16.65 | 16.28 | 8.98 | 4.50 | 3.75 | 4.79 | 17.96 | 0.100 | 0.100 | 0.140 | 0.140 | 0.042 | 0.000140 | 0.001 | 0.203 | 0.000147 | 0.0070 | 0.10 | 0.50 | 0.50 | 2.00 | 0.10 | 1.10 | 1.14 | 1.29 | 2.14 | 0.18 | 1.00 | 1.00 | 1.00 |
|  | 26 | Ghazvini | 16.49 | 16.49 | 3.75 | 8.98 | 4.50 | 3.75 | 5.19 | 17.96 | 0.502 | 0.501 | 0.140 | 0.140 | 0.042 | 0.000140 | 0.001 | 1.005 | 0.000147 | 0.0070 | 0.10 | 0.50 | 0.50 | 2.00 | 0.10 | 1.10 | 2.38 | 2.95 | 2.80 | 0.04 | 1.00 | 1.00 | 2.40 |
|  | 27 | Ghazvini | 16.49 | 16.49 | 0.33 | 0.75 | 0.38 | 0.31 | 1.13 | 1.50 | 0.234 | 0.234 | 0.401 | 0.400 | 0.120 | 0.000401 | 0.004 | 0.476 | 0.000420 | 0.0200 | 0.10 | 0.50 | 0.50 | 2.00 | 0.10 | 1.10 | 1.61 | 2.36 | 2.68 | 0.04 | 1.00 | 1.00 | 2.14 |
|  | 28 | Ghazvini | 22.68 | 28.94 | 7.74 | 3.49 | 1.75 | 1.46 | 2.77 | 6.98 | 0.502 | 0.501 | 0.401 | 0.400 | 0.120 | 0.000401 | 0.004 | 1.010 | 0.000420 | 0.0200 | 0.10 | 0.50 | 0.50 | 2.00 | 0.10 | 1.10 | 4.00 | 3.80 | 3.65 | 0.05 | 1.00 | 1.00 | 3.20 |
| **Control** | **POM** | **UCB1** | **19.14** | **29.36** | **10.85** | **2.59** | **1.90** | **1.81** | **4.08** | **0.48** | **0.112** | **0.112** | **0.078** | **0.186** | **0.035** | **0.000441** | **0.001** | **0.400** | **0.000084** | **0.0048** | **5.30** | **0.70** | **0.60** | **0.25** | **0.09** | **1.50** | **1.00** | **1.38** | **1.78** | **0.26** | **1.00** | **1.00** | **1.00** |
|  | **POM** | **Ghazvini** | **19.14** | **29.36** | **10.85** | **2.59** | **1.90** | **1.81** | **4.08** | **0.48** | **0.112** | **0.112** | **0.078** | **0.186** | **0.035** | **0.000441** | **0.001** | **0.400** | **0.000084** | **0.0048** | **5.30** | **0.70** | **0.60** | **0.25** | **0.09** | **1.50** | **1.52** | **1.28** | **1.22** | **0.21** | **1.00** | **1.06** | **1.00** |
|  | 1 | UCB1 | 28.71 | 43.45 | 15.61 | 3.56 | 2.61 | 2.49 | 5.84 | 0.66 | 0.091 | 0.091 | 0.212 | 0.507 | 0.097 | 0.001201 | 0.003 | 0.428 | 0.000229 | 0.0131 | 5.30 | 0.70 | 0.60 | 0.25 | 0.09 | 1.50 | 1.08 | 1.83 | 3.38 | 0.25 | 1.00 | 1.17 | 1.00 |
|  | 2 | UCB1 | 9.57 | 23.79 | 15.16 | 3.88 | 2.85 | 2.72 | 6.06 | 0.71 | 0.337 | 0.336 | 0.039 | 0.093 | 0.018 | 0.000220 | 0.001 | 0.934 | 0.000042 | 0.0024 | 5.30 | 0.70 | 0.60 | 0.25 | 0.09 | 1.50 | 1.00 | 1.36 | 2.03 | 0.09 | 1.00 | 2.12 | 1.41 |
|  | 3 | UCB1 | 28.71 | 33.82 | 5.42 | 1.29 | 0.95 | 0.91 | 2.04 | 0.24 | 0.056 | 0.056 | 0.039 | 0.093 | 0.018 | 0.000220 | 0.001 | 0.200 | 0.000042 | 0.0024 | 5.30 | 0.70 | 0.60 | 0.25 | 0.09 | 1.50 | 1.00 | 1.48 | 2.10 | 0.22 | 1.00 | 1.54 | 1.48 |
| **×POM medium** | 4 | UCB1 | 18.07 | 28.70 | 10.95 | 1.29 | 0.95 | 0.91 | 2.87 | 0.24 | 0.337 | 0.336 | 0.233 | 0.558 | 0.106 | 0.001322 | 0.004 | 0.767 | 0.000252 | 0.0145 | 5.30 | 0.70 | 0.60 | 0.25 | 0.09 | 1.50 | 1.11 | 1.22 | 2.55 | 0.18 | 1.00 | 3.06 | 1.00 |
|  | 5 | UCB1 | 26.32 | 32.08 | 6.08 | 1.29 | 0.95 | 0.91 | 2.35 | 0.24 | 0.302 | 0.301 | 0.063 | 0.151 | 0.029 | 0.000358 | 0.001 | 0.691 | 0.000068 | 0.0039 | 5.30 | 0.70 | 0.60 | 0.25 | 0.09 | 1.50 | 1.14 | 1.14 | 1.36 | 0.13 | 1.00 | 1.57 | 1.00 |
|  | 6 | UCB1 | 9.57 | 16.71 | 7.74 | 2.41 | 1.77 | 1.69 | 4.32 | 0.44 | 0.167 | 0.166 | 0.233 | 0.558 | 0.106 | 0.001322 | 0.004 | 0.502 | 0.000252 | 0.0145 | 5.30 | 0.70 | 0.60 | 0.25 | 0.09 | 1.50 | 1.00 | 1.67 | 1.69 | 0.27 | 1.00 | 1.28 | 1.00 |
|  | 7 | UCB1 | 11.96 | 21.55 | 10.50 | 3.76 | 2.76 | 2.63 | 5.90 | 0.69 | 0.323 | 0.322 | 0.048 | 0.115 | 0.022 | 0.000272 | 0.001 | 0.899 | 0.000052 | 0.0030 | 5.30 | 0.70 | 0.60 | 0.25 | 0.09 | 1.50 | 1.00 | 1.00 | 1.00 | 0.15 | 1.00 | 1.39 | 1.00 |
|  | 8 | UCB1 | 28.71 | 44.04 | 16.27 | 3.88 | 2.85 | 2.72 | 5.78 | 0.71 | 0.056 | 0.056 | 0.039 | 0.093 | 0.018 | 0.000220 | 0.001 | 0.374 | 0.000042 | 0.0024 | 5.30 | 0.70 | 0.60 | 0.25 | 0.09 | 1.50 | 1.22 | 1.89 | 2.89 | 0.22 | 1.00 | 1.61 | 1.61 |
|  | 9 | UCB1 | 9.57 | 16.67 | 7.41 | 1.29 | 0.95 | 0.91 | 2.31 | 0.24 | 0.056 | 0.056 | 0.133 | 0.319 | 0.061 | 0.000756 | 0.002 | 0.203 | 0.000144 | 0.0083 | 5.30 | 0.70 | 0.60 | 0.25 | 0.09 | 1.50 | 1.00 | 1.07 | 2.79 | 0.23 | 1.00 | 1.00 | 1.00 |
|  | 10 | UCB1 | 28.71 | 38.52 | 10.76 | 3.88 | 2.85 | 2.72 | 6.61 | 0.71 | 0.337 | 0.336 | 0.233 | 0.558 | 0.106 | 0.001322 | 0.004 | 0.940 | 0.000252 | 0.0145 | 5.30 | 0.70 | 0.60 | 0.25 | 0.09 | 1.50 | 1.10 | 1.03 | 1.15 | 0.22 | 1.00 | 1.35 | 1.00 |
|  | 11 | UCB1 | 14.44 | 25.63 | 12.14 | 3.88 | 2.85 | 2.72 | 6.22 | 0.71 | 0.056 | 0.056 | 0.192 | 0.459 | 0.088 | 0.001088 | 0.003 | 0.379 | 0.000208 | 0.0119 | 5.30 | 0.70 | 0.60 | 0.25 | 0.09 | 1.50 | 1.44 | 1.33 | 3.94 | 0.28 | 1.00 | 1.00 | 1.67 |
|  | 12 | UCB1 | 9.57 | 14.68 | 5.42 | 1.29 | 0.95 | 0.91 | 2.32 | 0.24 | 0.337 | 0.336 | 0.039 | 0.093 | 0.018 | 0.000220 | 0.001 | 0.760 | 0.000042 | 0.0024 | 5.30 | 0.70 | 0.60 | 0.25 | 0.09 | 1.50 | 1.00 | 1.19 | 1.63 | 0.11 | 1.00 | 1.13 | 1.00 |
|  | 13 | UCB1 | 28.71 | 39.34 | 10.94 | 1.29 | 0.95 | 0.91 | 2.14 | 0.24 | 0.158 | 0.158 | 0.039 | 0.093 | 0.018 | 0.000220 | 0.001 | 0.403 | 0.000042 | 0.0024 | 5.30 | 0.70 | 0.60 | 0.25 | 0.09 | 1.50 | 1.80 | 1.30 | 1.40 | 0.21 | 1.00 | 1.20 | 1.20 |
|  | 14 | UCB1 | 10.91 | 20.72 | 10.76 | 3.88 | 2.85 | 2.72 | 6.61 | 0.71 | 0.337 | 0.336 | 0.233 | 0.558 | 0.106 | 0.001322 | 0.004 | 0.940 | 0.000252 | 0.0145 | 5.30 | 0.70 | 0.60 | 0.25 | 0.09 | 1.50 | 1.00 | 1.00 | 1.06 | 0.19 | 1.00 | 1.31 | 1.00 |
|  | 15 | UCB1 | 9.57 | 22.41 | 13.45 | 2.51 | 1.84 | 1.76 | 4.38 | 0.46 | 0.337 | 0.336 | 0.143 | 0.343 | 0.065 | 0.000812 | 0.002 | 0.845 | 0.000155 | 0.0089 | 5.30 | 0.70 | 0.60 | 0.25 | 0.09 | 1.50 | 1.00 | 1.05 | 1.14 | 0.15 | 1.00 | 1.10 | 1.21 |
|  | 16 | UCB1 | 9.57 | 24.90 | 16.28 | 3.88 | 2.85 | 2.72 | 6.33 | 0.71 | 0.056 | 0.056 | 0.233 | 0.558 | 0.106 | 0.001322 | 0.004 | 0.380 | 0.000252 | 0.0145 | 5.30 | 0.70 | 0.60 | 0.25 | 0.09 | 1.50 | 1.33 | 1.75 | 3.72 | 0.20 | 1.00 | 1.51 | 1.03 |
|  | 17 | UCB1 | 28.71 | 33.82 | 5.44 | 1.29 | 0.95 | 0.91 | 2.87 | 0.24 | 0.337 | 0.336 | 0.233 | 0.558 | 0.106 | 0.001322 | 0.004 | 0.767 | 0.000252 | 0.0145 | 5.30 | 0.70 | 0.60 | 0.25 | 0.09 | 1.50 | 1.00 | 1.17 | 1.33 | 0.18 | 1.00 | 2.33 | 1.00 |
|  | 18 | UCB1 | 9.57 | 19.38 | 10.75 | 3.88 | 2.85 | 2.72 | 5.78 | 0.71 | 0.056 | 0.056 | 0.039 | 0.093 | 0.018 | 0.000220 | 0.001 | 0.374 | 0.000042 | 0.0024 | 5.30 | 0.70 | 0.60 | 0.25 | 0.09 | 1.50 | 1.21 | 1.34 | 2.54 | 0.20 | 1.00 | 1.29 | 1.00 |
|  | 19 | UCB1 | 23.93 | 33.72 | 10.56 | 3.11 | 2.29 | 2.18 | 5.18 | 0.57 | 0.219 | 0.218 | 0.159 | 0.382 | 0.073 | 0.000905 | 0.003 | 0.651 | 0.000173 | 0.0099 | 5.30 | 0.70 | 0.60 | 0.25 | 0.09 | 1.50 | 1.00 | 1.06 | 1.13 | 0.27 | 1.00 | 1.38 | 1.00 |
|  | 20 | UCB1 | 9.57 | 18.39 | 9.14 | 1.29 | 0.95 | 0.91 | 2.74 | 0.24 | 0.202 | 0.202 | 0.233 | 0.558 | 0.106 | 0.001322 | 0.004 | 0.498 | 0.000252 | 0.0145 | 5.30 | 0.70 | 0.60 | 0.25 | 0.09 | 1.50 | 1.00 | 2.14 | 1.50 | 0.21 | 1.00 | 1.00 | 1.14 |
|  | 21 | UCB1 | 28.11 | 42.16 | 14.91 | 3.56 | 2.61 | 2.49 | 5.63 | 0.65 | 0.337 | 0.336 | 0.054 | 0.129 | 0.025 | 0.000306 | 0.001 | 0.913 | 0.000058 | 0.0034 | 5.30 | 0.70 | 0.60 | 0.25 | 0.09 | 1.50 | 1.00 | 1.00 | 1.00 | 0.13 | 1.00 | 1.29 | 1.00 |
|  | 22 | UCB1 | 17.16 | 22.27 | 5.44 | 1.29 | 0.95 | 0.91 | 2.59 | 0.24 | 0.056 | 0.056 | 0.233 | 0.558 | 0.106 | 0.001322 | 0.004 | 0.206 | 0.000252 | 0.0145 | 5.30 | 0.70 | 0.60 | 0.25 | 0.09 | 1.50 | 1.29 | 1.93 | 3.89 | 0.22 | 1.00 | 1.14 | 1.00 |
|  | 23 | UCB1 | 11.07 | 20.14 | 9.66 | 2.43 | 1.78 | 1.70 | 4.05 | 0.45 | 0.227 | 0.227 | 0.106 | 0.253 | 0.048 | 0.000600 | 0.002 | 0.621 | 0.000114 | 0.0066 | 5.30 | 0.70 | 0.60 | 0.25 | 0.09 | 1.50 | 1.00 | 1.35 | 1.05 | 0.18 | 1.00 | 1.50 | 1.00 |
|  | 24 | UCB1 | 9.57 | 20.20 | 10.94 | 1.29 | 0.95 | 0.91 | 2.04 | 0.24 | 0.056 | 0.056 | 0.039 | 0.093 | 0.018 | 0.000220 | 0.001 | 0.200 | 0.000042 | 0.0024 | 5.30 | 0.70 | 0.60 | 0.25 | 0.09 | 1.50 | 1.00 | 1.66 | 1.31 | 0.29 | 1.00 | 1.10 | 1.25 |
|  | 25 | UCB1 | 26.32 | 36.23 | 10.77 | 3.56 | 2.61 | 2.49 | 5.31 | 0.65 | 0.056 | 0.056 | 0.039 | 0.093 | 0.018 | 0.000220 | 0.001 | 0.352 | 0.000042 | 0.0024 | 5.30 | 0.70 | 0.60 | 0.25 | 0.09 | 1.50 | 1.11 | 1.56 | 2.33 | 0.27 | 1.06 | 1.28 | 1.33 |
|  | 26 | UCB1 | 23.07 | 34.02 | 11.53 | 2.40 | 1.76 | 1.68 | 3.78 | 0.44 | 0.056 | 0.056 | 0.087 | 0.209 | 0.040 | 0.000496 | 0.001 | 0.276 | 0.000095 | 0.0054 | 5.30 | 0.70 | 0.60 | 0.25 | 0.09 | 1.50 | 1.00 | 1.28 | 2.22 | 0.28 | 1.33 | 1.78 | 1.00 |
|  | 27 | UCB1 | 28.71 | 39.34 | 10.95 | 1.29 | 0.95 | 0.91 | 2.59 | 0.24 | 0.056 | 0.056 | 0.233 | 0.558 | 0.106 | 0.001322 | 0.004 | 0.206 | 0.000252 | 0.0145 | 5.30 | 0.70 | 0.60 | 0.25 | 0.09 | 1.50 | 1.50 | 1.33 | 1.83 | 0.37 | 1.00 | 1.17 | 1.00 |
|  | 28 | UCB1 | 28.71 | 38.52 | 10.76 | 3.88 | 2.85 | 2.72 | 6.33 | 0.71 | 0.056 | 0.056 | 0.233 | 0.558 | 0.106 | 0.001322 | 0.004 | 0.380 | 0.000252 | 0.0145 | 5.30 | 0.70 | 0.60 | 0.25 | 0.09 | 1.50 | 1.00 | 1.00 | 3.70 | 0.16 | 1.00 | 1.00 | 1.40 |
|  | 29 | UCB1 | 28.71 | 44.04 | 16.28 | 3.88 | 2.85 | 2.72 | 6.61 | 0.71 | 0.337 | 0.336 | 0.233 | 0.558 | 0.106 | 0.001322 | 0.004 | 0.940 | 0.000252 | 0.0145 | 5.30 | 0.70 | 0.60 | 0.25 | 0.09 | 1.50 | 1.00 | 1.22 | 1.00 | 0.17 | 1.00 | 1.38 | 1.00 |
|  | 30 | UCB1 | 16.23 | 31.56 | 16.27 | 3.88 | 2.85 | 2.72 | 6.16 | 0.71 | 0.197 | 0.197 | 0.123 | 0.294 | 0.056 | 0.000696 | 0.002 | 0.658 | 0.000133 | 0.0076 | 5.30 | 0.70 | 0.60 | 0.25 | 0.09 | 1.50 | 1.00 | 1.03 | 1.00 | 0.24 | 1.00 | 1.17 | 1.00 |
|  | 31 | UCB1 | 28.71 | 38.52 | 10.75 | 3.88 | 2.85 | 2.72 | 6.06 | 0.71 | 0.337 | 0.336 | 0.039 | 0.093 | 0.018 | 0.000220 | 0.001 | 0.934 | 0.000042 | 0.0024 | 5.30 | 0.70 | 0.60 | 0.25 | 0.09 | 1.50 | 1.00 | 1.00 | 1.00 | 0.07 | 1.00 | 1.00 | 1.00 |
|  | 32 | UCB1 | 9.57 | 14.68 | 5.44 | 1.29 | 0.95 | 0.91 | 2.87 | 0.24 | 0.337 | 0.336 | 0.233 | 0.558 | 0.106 | 0.001322 | 0.004 | 0.767 | 0.000252 | 0.0145 | 5.30 | 0.70 | 0.60 | 0.25 | 0.09 | 1.50 | 1.25 | 1.00 | 1.38 | 0.20 | 1.00 | 1.13 | 1.00 |
|  | 33 | UCB1 | 28.71 | 39.34 | 10.94 | 1.29 | 0.95 | 0.91 | 2.32 | 0.24 | 0.337 | 0.336 | 0.039 | 0.093 | 0.018 | 0.000220 | 0.001 | 0.760 | 0.000042 | 0.0024 | 5.30 | 0.70 | 0.60 | 0.25 | 0.09 | 1.50 | 1.00 | 1.00 | 2.43 | 0.14 | 1.00 | 1.71 | 1.00 |
|  | 1 | Ghazvini | 28.71 | 43.45 | 15.61 | 3.56 | 2.61 | 2.49 | 5.84 | 0.66 | 0.091 | 0.091 | 0.212 | 0.507 | 0.097 | 0.001201 | 0.003 | 0.428 | 0.000229 | 0.0131 | 5.30 | 0.70 | 0.60 | 0.25 | 0.09 | 1.50 | 1.20 | 1.80 | 1.80 | 0.22 | 1.00 | 1.30 | 1.00 |
|  | 2 | Ghazvini | 9.57 | 23.79 | 15.16 | 3.88 | 2.85 | 2.72 | 6.06 | 0.71 | 0.337 | 0.336 | 0.039 | 0.093 | 0.018 | 0.000220 | 0.001 | 0.934 | 0.000042 | 0.0024 | 5.30 | 0.70 | 0.60 | 0.25 | 0.09 | 1.50 | 1.00 | 1.03 | 1.10 | 0.06 | 1.00 | 1.48 | 1.00 |
|  | 3 | Ghazvini | 28.71 | 33.82 | 5.42 | 1.29 | 0.95 | 0.91 | 2.04 | 0.24 | 0.056 | 0.056 | 0.039 | 0.093 | 0.018 | 0.000220 | 0.001 | 0.200 | 0.000042 | 0.0024 | 5.30 | 0.70 | 0.60 | 0.25 | 0.09 | 1.50 | 1.50 | 1.98 | 1.16 | 0.16 | 1.00 | 1.54 | 1.13 |
|  | 4 | Ghazvini | 18.07 | 28.70 | 10.95 | 1.29 | 0.95 | 0.91 | 2.87 | 0.24 | 0.337 | 0.336 | 0.233 | 0.558 | 0.106 | 0.001322 | 0.004 | 0.767 | 0.000252 | 0.0145 | 5.30 | 0.70 | 0.60 | 0.25 | 0.09 | 1.50 | 1.41 | 1.28 | 1.31 | 0.17 | 1.00 | 1.78 | 1.00 |
|  | 5 | Ghazvini | 26.32 | 32.08 | 6.08 | 1.29 | 0.95 | 0.91 | 2.35 | 0.24 | 0.302 | 0.301 | 0.063 | 0.151 | 0.029 | 0.000358 | 0.001 | 0.691 | 0.000068 | 0.0039 | 5.30 | 0.70 | 0.60 | 0.25 | 0.09 | 1.50 | 1.39 | 1.50 | 1.50 | 0.13 | 1.00 | 2.94 | 1.00 |
|  | 6 | Ghazvini | 9.57 | 16.71 | 7.74 | 2.41 | 1.77 | 1.69 | 4.32 | 0.44 | 0.167 | 0.166 | 0.233 | 0.558 | 0.106 | 0.001322 | 0.004 | 0.502 | 0.000252 | 0.0145 | 5.30 | 0.70 | 0.60 | 0.25 | 0.09 | 1.50 | 1.30 | 1.30 | 1.00 | 0.22 | 1.00 | 1.38 | 1.00 |
|  | 7 | Ghazvini | 11.96 | 21.55 | 10.50 | 3.76 | 2.76 | 2.63 | 5.90 | 0.69 | 0.323 | 0.322 | 0.048 | 0.115 | 0.022 | 0.000272 | 0.001 | 0.899 | 0.000052 | 0.0030 | 5.30 | 0.70 | 0.60 | 0.25 | 0.09 | 1.50 | 1.00 | 1.00 | 1.00 | 0.11 | 1.00 | 1.63 | 1.00 |
|  | 8 | Ghazvini | 28.71 | 44.04 | 16.27 | 3.88 | 2.85 | 2.72 | 5.78 | 0.71 | 0.056 | 0.056 | 0.039 | 0.093 | 0.018 | 0.000220 | 0.001 | 0.374 | 0.000042 | 0.0024 | 5.30 | 0.70 | 0.60 | 0.25 | 0.09 | 1.50 | 1.40 | 1.83 | 1.10 | 0.20 | 1.00 | 1.13 | 1.17 |
|  | 9 | Ghazvini | 9.57 | 16.67 | 7.41 | 1.29 | 0.95 | 0.91 | 2.31 | 0.24 | 0.056 | 0.056 | 0.133 | 0.319 | 0.061 | 0.000756 | 0.002 | 0.203 | 0.000144 | 0.0083 | 5.30 | 0.70 | 0.60 | 0.25 | 0.09 | 1.50 | 1.00 | 1.60 | 1.60 | 0.18 | 1.00 | 1.40 | 1.00 |
|  | 10 | Ghazvini | 28.71 | 38.52 | 10.76 | 3.88 | 2.85 | 2.72 | 6.61 | 0.71 | 0.337 | 0.336 | 0.233 | 0.558 | 0.106 | 0.001322 | 0.004 | 0.940 | 0.000252 | 0.0145 | 5.30 | 0.70 | 0.60 | 0.25 | 0.09 | 1.50 | 1.60 | 1.20 | 1.00 | 0.18 | 1.00 | 1.10 | 1.00 |
|  | 11 | Ghazvini | 14.44 | 25.63 | 12.14 | 3.88 | 2.85 | 2.72 | 6.22 | 0.71 | 0.056 | 0.056 | 0.192 | 0.459 | 0.088 | 0.001088 | 0.003 | 0.379 | 0.000208 | 0.0119 | 5.30 | 0.70 | 0.60 | 0.25 | 0.09 | 1.50 | 1.78 | 1.94 | 2.50 | 0.19 | 1.00 | 1.11 | 1.11 |
|  | 12 | Ghazvini | 9.57 | 14.68 | 5.42 | 1.29 | 0.95 | 0.91 | 2.32 | 0.24 | 0.337 | 0.336 | 0.039 | 0.093 | 0.018 | 0.000220 | 0.001 | 0.760 | 0.000042 | 0.0024 | 5.30 | 0.70 | 0.60 | 0.25 | 0.09 | 1.50 | 1.00 | 1.13 | 1.10 | 0.07 | 1.00 | 1.13 | 1.00 |
|  | 13 | Ghazvini | 28.71 | 39.34 | 10.94 | 1.29 | 0.95 | 0.91 | 2.14 | 0.24 | 0.158 | 0.158 | 0.039 | 0.093 | 0.018 | 0.000220 | 0.001 | 0.403 | 0.000042 | 0.0024 | 5.30 | 0.70 | 0.60 | 0.25 | 0.09 | 1.50 | 1.33 | 1.56 | 1.44 | 0.19 | 1.00 | 2.50 | 1.00 |
|  | 14 | Ghazvini | 10.91 | 20.72 | 10.76 | 3.88 | 2.85 | 2.72 | 6.61 | 0.71 | 0.337 | 0.336 | 0.233 | 0.558 | 0.106 | 0.001322 | 0.004 | 0.940 | 0.000252 | 0.0145 | 5.30 | 0.70 | 0.60 | 0.25 | 0.09 | 1.50 | 1.00 | 1.06 | 1.00 | 0.13 | 1.00 | 1.94 | 1.00 |
|  | 15 | Ghazvini | 9.57 | 22.41 | 13.45 | 2.51 | 1.84 | 1.76 | 4.38 | 0.46 | 0.337 | 0.336 | 0.143 | 0.343 | 0.065 | 0.000812 | 0.002 | 0.845 | 0.000155 | 0.0089 | 5.30 | 0.70 | 0.60 | 0.25 | 0.09 | 1.50 | 1.07 | 1.11 | 1.00 | 0.09 | 1.00 | 1.21 | 1.00 |
|  | 16 | Ghazvini | 9.57 | 24.90 | 16.28 | 3.88 | 2.85 | 2.72 | 6.33 | 0.71 | 0.056 | 0.056 | 0.233 | 0.558 | 0.106 | 0.001322 | 0.004 | 0.380 | 0.000252 | 0.0145 | 5.30 | 0.70 | 0.60 | 0.25 | 0.09 | 1.50 | 1.15 | 1.53 | 2.03 | 0.13 | 1.00 | 1.65 | 1.00 |
|  | 17 | Ghazvini | 28.71 | 33.82 | 5.44 | 1.29 | 0.95 | 0.91 | 2.87 | 0.24 | 0.337 | 0.336 | 0.233 | 0.558 | 0.106 | 0.001322 | 0.004 | 0.767 | 0.000252 | 0.0145 | 5.30 | 0.70 | 0.60 | 0.25 | 0.09 | 1.50 | 1.00 | 1.00 | 1.19 | 0.12 | 1.00 | 1.44 | 1.00 |
|  | 18 | Ghazvini | 9.57 | 19.38 | 10.75 | 3.88 | 2.85 | 2.72 | 5.78 | 0.71 | 0.056 | 0.056 | 0.039 | 0.093 | 0.018 | 0.000220 | 0.001 | 0.374 | 0.000042 | 0.0024 | 5.30 | 0.70 | 0.60 | 0.25 | 0.09 | 1.50 | 1.05 | 1.21 | 1.65 | 0.17 | 1.15 | 1.53 | 1.00 |
|  | 19 | Ghazvini | 23.93 | 33.72 | 10.56 | 3.11 | 2.29 | 2.18 | 5.18 | 0.57 | 0.219 | 0.218 | 0.159 | 0.382 | 0.073 | 0.000905 | 0.003 | 0.651 | 0.000173 | 0.0099 | 5.30 | 0.70 | 0.60 | 0.25 | 0.09 | 1.50 | 1.90 | 1.30 | 1.00 | 0.26 | 1.00 | 1.25 | 1.00 |
|  | 20 | Ghazvini | 9.57 | 18.39 | 9.14 | 1.29 | 0.95 | 0.91 | 2.74 | 0.24 | 0.202 | 0.202 | 0.233 | 0.558 | 0.106 | 0.001322 | 0.004 | 0.498 | 0.000252 | 0.0145 | 5.30 | 0.70 | 0.60 | 0.25 | 0.09 | 1.50 | 1.00 | 1.06 | 1.17 | 0.18 | 1.00 | 1.44 | 1.00 |
|  | 21 | Ghazvini | 28.11 | 42.16 | 14.91 | 3.56 | 2.61 | 2.49 | 5.63 | 0.65 | 0.337 | 0.336 | 0.054 | 0.129 | 0.025 | 0.000306 | 0.001 | 0.913 | 0.000058 | 0.0034 | 5.30 | 0.70 | 0.60 | 0.25 | 0.09 | 1.50 | 1.00 | 1.00 | 1.00 | 0.08 | 1.00 | 1.00 | 1.00 |
|  | 22 | Ghazvini | 17.16 | 22.27 | 5.44 | 1.29 | 0.95 | 0.91 | 2.59 | 0.24 | 0.056 | 0.056 | 0.233 | 0.558 | 0.106 | 0.001322 | 0.004 | 0.206 | 0.000252 | 0.0145 | 5.30 | 0.70 | 0.60 | 0.25 | 0.09 | 1.50 | 1.00 | 1.22 | 1.94 | 0.18 | 1.33 | 1.33 | 1.00 |
|  | 23 | Ghazvini | 11.07 | 20.14 | 9.66 | 2.43 | 1.78 | 1.70 | 4.05 | 0.45 | 0.227 | 0.227 | 0.106 | 0.253 | 0.048 | 0.000600 | 0.002 | 0.621 | 0.000114 | 0.0066 | 5.30 | 0.70 | 0.60 | 0.25 | 0.09 | 1.50 | 1.14 | 1.07 | 1.00 | 0.16 | 1.00 | 1.00 | 1.00 |
|  | 24 | Ghazvini | 9.57 | 20.20 | 10.94 | 1.29 | 0.95 | 0.91 | 2.04 | 0.24 | 0.056 | 0.056 | 0.039 | 0.093 | 0.018 | 0.000220 | 0.001 | 0.200 | 0.000042 | 0.0024 | 5.30 | 0.70 | 0.60 | 0.25 | 0.09 | 1.50 | 1.00 | 1.30 | 1.20 | 0.19 | 1.00 | 1.28 | 1.00 |
|  | 25 | Ghazvini | 26.32 | 36.23 | 10.77 | 3.56 | 2.61 | 2.49 | 5.31 | 0.65 | 0.056 | 0.056 | 0.039 | 0.093 | 0.018 | 0.000220 | 0.001 | 0.352 | 0.000042 | 0.0024 | 5.30 | 0.70 | 0.60 | 0.25 | 0.09 | 1.50 | 1.44 | 1.22 | 1.06 | 0.16 | 1.00 | 1.33 | 1.00 |
|  | 26 | Ghazvini | 23.07 | 34.02 | 11.53 | 2.40 | 1.76 | 1.68 | 3.78 | 0.44 | 0.056 | 0.056 | 0.087 | 0.209 | 0.040 | 0.000496 | 0.001 | 0.276 | 0.000095 | 0.0054 | 5.30 | 0.70 | 0.60 | 0.25 | 0.09 | 1.50 | 1.40 | 1.45 | 1.35 | 0.22 | 1.00 | 1.95 | 1.10 |
|  | 27 | Ghazvini | 28.71 | 39.34 | 10.95 | 1.29 | 0.95 | 0.91 | 2.59 | 0.24 | 0.056 | 0.056 | 0.233 | 0.558 | 0.106 | 0.001322 | 0.004 | 0.206 | 0.000252 | 0.0145 | 5.30 | 0.70 | 0.60 | 0.25 | 0.09 | 1.50 | 1.63 | 1.56 | 1.81 | 0.22 | 1.19 | 1.25 | 1.00 |
|  | 28 | Ghazvini | 28.71 | 38.52 | 10.76 | 3.88 | 2.85 | 2.72 | 6.33 | 0.71 | 0.056 | 0.056 | 0.233 | 0.558 | 0.106 | 0.001322 | 0.004 | 0.380 | 0.000252 | 0.0145 | 5.30 | 0.70 | 0.60 | 0.25 | 0.09 | 1.50 | 2.43 | 1.43 | 1.43 | 0.19 | 1.00 | 1.00 | 1.21 |
|  | 29 | Ghazvini | 28.71 | 44.04 | 16.28 | 3.88 | 2.85 | 2.72 | 6.61 | 0.71 | 0.337 | 0.336 | 0.233 | 0.558 | 0.106 | 0.001322 | 0.004 | 0.940 | 0.000252 | 0.0145 | 5.30 | 0.70 | 0.60 | 0.25 | 0.09 | 1.50 | 1.06 | 1.28 | 1.00 | 0.16 | 1.00 | 1.09 | 1.00 |
|  | 30 | Ghazvini | 16.23 | 31.56 | 16.27 | 3.88 | 2.85 | 2.72 | 6.16 | 0.71 | 0.197 | 0.197 | 0.123 | 0.294 | 0.056 | 0.000696 | 0.002 | 0.658 | 0.000133 | 0.0076 | 5.30 | 0.70 | 0.60 | 0.25 | 0.09 | 1.50 | 1.15 | 1.14 | 1.00 | 0.17 | 1.00 | 1.03 | 1.00 |
|  | 31 | Ghazvini | 28.71 | 38.52 | 10.75 | 3.88 | 2.85 | 2.72 | 6.06 | 0.71 | 0.337 | 0.336 | 0.039 | 0.093 | 0.018 | 0.000220 | 0.001 | 0.934 | 0.000042 | 0.0024 | 5.30 | 0.70 | 0.60 | 0.25 | 0.09 | 1.50 | 1.00 | 1.00 | 1.00 | 0.09 | 1.00 | 1.50 | 1.00 |
|  | 32 | Ghazvini | 9.57 | 14.68 | 5.44 | 1.29 | 0.95 | 0.91 | 2.87 | 0.24 | 0.337 | 0.336 | 0.233 | 0.558 | 0.106 | 0.001322 | 0.004 | 0.767 | 0.000252 | 0.0145 | 5.30 | 0.70 | 0.60 | 0.25 | 0.09 | 1.50 | 1.44 | 1.44 | 1.22 | 0.14 | 1.00 | 1.11 | 1.00 |
|  | 33 | Ghazvini | 28.71 | 39.34 | 10.94 | 1.29 | 0.95 | 0.91 | 2.32 | 0.24 | 0.337 | 0.336 | 0.039 | 0.093 | 0.018 | 0.000220 | 0.001 | 0.760 | 0.000042 | 0.0024 | 5.30 | 0.70 | 0.60 | 0.25 | 0.09 | 1.50 | 1.67 | 1.33 | 1.00 | 0.16 | 1.00 | 1.75 | 1.08 |
|  | **Min.** |  | **4.12** | **4.12** | **0.31** | **0.75** | **0.38** | **0.31** | **0.49** | **0.24** | **0.06** | **0.06** | **0.01** | **0.01** | **0.00** | **0.000000** | **0.000** | **0.20** | **0.000000** | **0.00** | **0.10** | **0.50** | **0.00** | **0.25** | **0.09** | **1.10** | **1.00** | **1.00** | **1.00** | **0.00** | **1.00** | **1.00** | **1.00** |
|  | **Max.** |  | **28.71** | **44.04** | **22.56** | **9.30** | **4.50** | **3.75** | **12.33** | **17.96** | **0.50** | **0.50** | **0.40** | **0.56** | **0.12** | **0.001321** | **0.004** | **1.01** | 0.000420 | **0.02** | **5.30** | **1.00** | **0.60** | **2.00** | **0.10** | **1.50** | **4.00** | **4.00** | **4.00** | **0.37** | **1.33** | **3.06** | **3.89** |

**TABLE S2|** Mineral compositions of the different culture media based on five-factor design space, vitamin-mixture and PGRs to alleviate physiological disorders of pistachio rootstocks. Data for STN and BC for MS medium come from previous dataset^51^.

|  | Treatment N0. | Genotype | Minerals  (mg L^-1^) | | | | | | | | | | | | | | | | | Vitamins  (mg L^-1^) | | | | PGRs  (mg L^-1^) | |
| --- | --- | --- | --- | --- | --- | --- | --- | --- | --- | --- | --- | --- | --- | --- | --- | --- | --- | --- | --- | --- | --- | --- | --- | --- | --- |
|  |  |  | KNO_3_ | NH4NO_3_ | Ca(NO_3_)_2_ • 4H_2_O | CaCl_2_ •2H_2_O | MgSO_4_ •7H_2_O | KH_2_PO_4_ | K2SO_4_ | NaH_2_PO_4_ • H_2_O | MnSO_4_ • 4H_2_O | ZnSO_4_ • 7H_2_O | H3BO_3_ | KI | CuSO_4_ • 5H_2_O | Na_2_MoO_4_ • 2H_2_O | CoCl_2_ • 6H_2_O | FeSO_4_ • 7H_2_O | Na_2_ EDTA • 2H_2_O | Thiamine-HCl | Nicotinic Acid | Pyridoxine-HCl | Glycine | IBA | BA |
| **Control** | **MS** | **UCB1** | **1900.00** | **1650.00** | **0.00** | **440.00** | **370.00** | **170.00** | **0.00** | **0.00** | **22.30** | **8.60** | **6.20** | **0.83** | **0.025** | **0.250** | **0.025** | **27.90** | **37.30** | **0.10** | **0.50** | **0.50** | **2.00** | **0.10** | **1.10** |
|  | **WPM** | **UCB1** | **0.00** | **400.00** | **556.00** | **96.00** | **370.00** | **170.00** | **990.00** | **0.00** | **29.42** | **8.60** | **6.20** | **0.00** | **0.250** | **0.250** | **0.000** | **27.90** | **37.30** | **1.00** | **0.50** | **0.50** | **2.00** | **0.10** | **1.10** |
|  | **DKW** | **UCB1** | **0.00** | **1417.00** | **1960.00** | **147.00** | **740.00** | **229.00** | **1559.00** | **0.00** | **44.59** | **16.45** | **4.80** | **0.00** | **0.250** | **0.387** | **0.000** | **33.36** | **44.67** | **2.00** | **1.00** | **0.00** | **2.00** | **0.10** | **1.10** |
|  | **MS** | **Ghazvini** | **1900.00** | **1650.00** | **0.00** | **440.00** | **370.00** | **170.00** | **0.00** | **0.00** | **22.30** | **8.60** | **6.20** | **0.83** | **0.025** | **0.250** | **0.025** | **27.90** | **37.30** | **0.10** | **0.50** | **0.50** | **2.00** | **0.10** | **1.10** |
|  | **WPM** | **Ghazvini** | **0.00** | **400.00** | **556.00** | **96.00** | **370.00** | **170.00** | **990.00** | **0.00** | **29.42** | **8.60** | **6.20** | **0.00** | **0.250** | **0.250** | **0.000** | **27.90** | **37.30** | **1.00** | **0.50** | **0.50** | **2.00** | **0.10** | **1.10** |
|  | **DKW** | **Ghazvini** | **0.00** | **1417.00** | **1960.00** | **147.00** | **740.00** | **229.00** | **1559.00** | **0.00** | **44.59** | **16.45** | **4.80** | **0.00** | **0.250** | **0.387** | **0.000** | **33.36** | **44.67** | **2.00** | **1.00** | **0.00** | **2.00** | **0.10** | **1.10** |
| ×MS Medium | 1 | UCB1 | 1900.00 | 330.00 | 0.00 | 110.00 | 92.50 | 42.50 | 0.00 | 0.00 | 89.20 | 34.40 | 24.80 | 3.32 | 0.100 | 1.000 | 0.100 | 65.10 | 87.03 | 0.10 | 0.50 | 0.50 | 2.00 | 0.10 | 1.10 |
|  | 2 | UCB1 | 0.00 | 330.00 | 0.00 | 916.67 | 770.83 | 354.17 | 0.00 | 0.00 | 89.20 | 34.40 | 24.80 | 3.32 | 0.100 | 1.000 | 0.100 | 27.90 | 37.30 | 0.10 | 0.50 | 0.50 | 2.00 | 0.10 | 1.10 |
|  | 3 | UCB1 | 1900.00 | 1815.00 | 0.00 | 916.67 | 770.83 | 354.17 | 0.00 | 0.00 | 2.23 | 0.86 | 0.62 | 0.08 | 0.003 | 0.025 | 0.003 | 27.90 | 37.30 | 0.10 | 0.50 | 0.50 | 2.00 | 0.10 | 1.10 |
|  | 4 | UCB1 | 0.00 | 1815.00 | 0.00 | 110.00 | 92.50 | 42.50 | 0.00 | 0.00 | 31.22 | 12.04 | 8.68 | 1.16 | 0.035 | 0.350 | 0.035 | 102.30 | 136.77 | 0.10 | 0.50 | 0.50 | 2.00 | 0.10 | 1.10 |
|  | 5 | UCB1 | 0.00 | 1815.00 | 0.00 | 1320.00 | 1110.00 | 510.00 | 0.00 | 0.00 | 89.20 | 34.40 | 24.80 | 3.32 | 0.100 | 1.000 | 0.100 | 27.90 | 37.30 | 0.10 | 0.50 | 0.50 | 2.00 | 0.10 | 1.10 |
|  | 6 | UCB1 | 950.00 | 330.00 | 0.00 | 1320.00 | 1110.00 | 510.00 | 0.00 | 0.00 | 2.23 | 0.86 | 0.62 | 0.08 | 0.003 | 0.025 | 0.003 | 139.50 | 186.50 | 0.10 | 0.50 | 0.50 | 2.00 | 0.10 | 1.10 |
|  | 7 | UCB1 | 0.00 | 330.00 | 0.00 | 110.00 | 92.50 | 42.50 | 0.00 | 0.00 | 2.23 | 0.86 | 0.62 | 0.08 | 0.003 | 0.025 | 0.003 | 27.90 | 37.30 | 0.10 | 0.50 | 0.50 | 2.00 | 0.10 | 1.10 |
|  | 8 | UCB1 | 0.00 | 1815.00 | 0.00 | 1320.00 | 1110.00 | 510.00 | 0.00 | 0.00 | 89.20 | 34.40 | 24.80 | 3.32 | 0.100 | 1.000 | 0.100 | 139.50 | 186.50 | 0.10 | 0.50 | 0.50 | 2.00 | 0.10 | 1.10 |
|  | 9 | UCB1 | 633.33 | 825.00 | 0.00 | 110.00 | 92.50 | 42.50 | 0.00 | 0.00 | 2.23 | 0.86 | 0.62 | 0.08 | 0.003 | 0.025 | 0.003 | 139.50 | 186.50 | 0.10 | 0.50 | 0.50 | 2.00 | 0.10 | 1.10 |
|  | 10 | UCB1 | 0.00 | 825.00 | 0.00 | 1320.00 | 1110.00 | 510.00 | 0.00 | 0.00 | 2.23 | 0.86 | 0.62 | 0.08 | 0.003 | 0.025 | 0.003 | 65.10 | 87.03 | 0.10 | 0.50 | 0.50 | 2.00 | 0.10 | 1.10 |
|  | 11 | UCB1 | 1900.00 | 1815.00 | 0.00 | 110.00 | 92.50 | 42.50 | 0.00 | 0.00 | 89.20 | 34.40 | 24.80 | 3.32 | 0.100 | 1.000 | 0.100 | 27.90 | 37.30 | 0.10 | 0.50 | 0.50 | 2.00 | 0.10 | 1.10 |
|  | 12 | UCB1 | 0.00 | 330.00 | 0.00 | 513.33 | 431.67 | 198.33 | 0.00 | 0.00 | 60.21 | 23.22 | 16.74 | 2.24 | 0.068 | 0.675 | 0.068 | 139.50 | 186.50 | 0.10 | 0.50 | 0.50 | 2.00 | 0.10 | 1.10 |
|  | 13 | UCB1 | 1900.00 | 1072.50 | 0.00 | 110.00 | 92.50 | 42.50 | 0.00 | 0.00 | 2.23 | 0.86 | 0.62 | 0.08 | 0.003 | 0.025 | 0.003 | 27.90 | 37.30 | 0.10 | 0.50 | 0.50 | 2.00 | 0.10 | 1.10 |
|  | 14 | UCB1 | 633.33 | 330.00 | 0.00 | 1320.00 | 1110.00 | 510.00 | 0.00 | 0.00 | 89.20 | 34.40 | 24.80 | 3.32 | 0.100 | 1.000 | 0.100 | 102.30 | 136.77 | 0.10 | 0.50 | 0.50 | 2.00 | 0.10 | 1.10 |
|  | 15 | UCB1 | 1900.00 | 330.00 | 0.00 | 513.33 | 431.67 | 198.33 | 0.00 | 0.00 | 2.23 | 0.86 | 0.62 | 0.08 | 0.003 | 0.025 | 0.003 | 102.30 | 136.77 | 0.10 | 0.50 | 0.50 | 2.00 | 0.10 | 1.10 |
|  | 16 | UCB1 | 1266.67 | 1815.00 | 0.00 | 1320.00 | 1110.00 | 510.00 | 0.00 | 0.00 | 2.23 | 0.86 | 0.62 | 0.08 | 0.003 | 0.025 | 0.003 | 102.30 | 136.77 | 0.10 | 0.50 | 0.50 | 2.00 | 0.10 | 1.10 |
|  | 17 | UCB1 | 0.00 | 1815.00 | 0.00 | 715.00 | 601.25 | 276.25 | 0.00 | 0.00 | 2.23 | 0.86 | 0.62 | 0.08 | 0.003 | 0.025 | 0.003 | 27.90 | 37.30 | 0.10 | 0.50 | 0.50 | 2.00 | 0.10 | 1.10 |
|  | 18 | UCB1 | 1900.00 | 1320.00 | 0.00 | 1320.00 | 1110.00 | 510.00 | 0.00 | 0.00 | 89.20 | 34.40 | 24.80 | 3.32 | 0.100 | 1.000 | 0.100 | 65.10 | 87.03 | 0.10 | 0.50 | 0.50 | 2.00 | 0.10 | 1.10 |
|  | 19 | UCB1 | 1900.00 | 1320.00 | 0.00 | 110.00 | 92.50 | 42.50 | 0.00 | 0.00 | 60.21 | 23.22 | 16.74 | 2.24 | 0.068 | 0.675 | 0.068 | 139.50 | 186.50 | 0.10 | 0.50 | 0.50 | 2.00 | 0.10 | 1.10 |
|  | 20 | UCB1 | 1900.00 | 1815.00 | 0.00 | 110.00 | 92.50 | 42.50 | 0.00 | 0.00 | 2.23 | 0.86 | 0.62 | 0.08 | 0.003 | 0.025 | 0.003 | 102.30 | 136.77 | 0.10 | 0.50 | 0.50 | 2.00 | 0.10 | 1.10 |
|  | 21 | UCB1 | 950.00 | 1072.50 | 0.00 | 715.00 | 601.25 | 276.25 | 0.00 | 0.00 | 45.72 | 17.63 | 12.71 | 1.70 | 0.051 | 0.513 | 0.051 | 83.70 | 111.90 | 0.10 | 0.50 | 0.50 | 2.00 | 0.10 | 1.10 |
|  | 22 | UCB1 | 1900.00 | 1815.00 | 0.00 | 1320.00 | 1110.00 | 510.00 | 0.00 | 0.00 | 60.21 | 23.22 | 16.74 | 2.24 | 0.068 | 0.675 | 0.068 | 139.50 | 186.50 | 0.10 | 0.50 | 0.50 | 2.00 | 0.10 | 1.10 |
|  | 23 | UCB1 | 1900.00 | 330.00 | 0.00 | 1320.00 | 1110.00 | 510.00 | 0.00 | 0.00 | 60.21 | 23.22 | 16.74 | 2.24 | 0.068 | 0.675 | 0.068 | 139.50 | 186.50 | 0.10 | 0.50 | 0.50 | 2.00 | 0.10 | 1.10 |
|  | 24 | UCB1 | 950.00 | 1072.50 | 0.00 | 715.00 | 601.25 | 276.25 | 0.00 | 0.00 | 45.72 | 17.63 | 12.71 | 1.70 | 0.051 | 0.513 | 0.051 | 55.80 | 74.60 | 0.10 | 0.50 | 0.50 | 2.00 | 0.10 | 1.10 |
|  | 25 | UCB1 | 1266.67 | 330.00 | 0.00 | 1320.00 | 1110.00 | 510.00 | 0.00 | 0.00 | 31.22 | 12.04 | 8.68 | 1.16 | 0.035 | 0.350 | 0.035 | 27.90 | 37.30 | 0.10 | 0.50 | 0.50 | 2.00 | 0.10 | 1.10 |
|  | 26 | UCB1 | 0.00 | 1320.00 | 0.00 | 1320.00 | 1110.00 | 510.00 | 0.00 | 0.00 | 31.22 | 12.04 | 8.68 | 1.16 | 0.035 | 0.350 | 0.035 | 139.50 | 186.50 | 0.10 | 0.50 | 0.50 | 2.00 | 0.10 | 1.10 |
|  | 27 | UCB1 | 0.00 | 1320.00 | 0.00 | 110.00 | 92.50 | 42.50 | 0.00 | 0.00 | 89.20 | 34.40 | 24.80 | 3.32 | 0.100 | 1.000 | 0.100 | 65.10 | 87.03 | 0.10 | 0.50 | 0.50 | 2.00 | 0.10 | 1.10 |
|  | 28 | UCB1 | 633.33 | 1815.00 | 0.00 | 513.33 | 431.67 | 198.33 | 0.00 | 0.00 | 89.20 | 34.40 | 24.80 | 3.32 | 0.100 | 1.000 | 0.100 | 139.50 | 186.50 | 0.10 | 0.50 | 0.50 | 2.00 | 0.10 | 1.10 |
|  | 1 | Ghazvini | 1900.00 | 330.00 | 0.00 | 110.00 | 92.50 | 42.50 | 0.00 | 0.00 | 89.20 | 34.40 | 24.80 | 3.32 | 0.100 | 1.000 | 0.100 | 65.10 | 87.03 | 0.10 | 0.50 | 0.50 | 2.00 | 0.10 | 1.10 |
|  | 2 | Ghazvini | 0.00 | 330.00 | 0.00 | 916.67 | 770.83 | 354.17 | 0.00 | 0.00 | 89.20 | 34.40 | 24.80 | 3.32 | 0.100 | 1.000 | 0.100 | 27.90 | 37.30 | 0.10 | 0.50 | 0.50 | 2.00 | 0.10 | 1.10 |
|  | 3 | Ghazvini | 1900.00 | 1815.00 | 0.00 | 916.67 | 770.83 | 354.17 | 0.00 | 0.00 | 2.23 | 0.86 | 0.62 | 0.08 | 0.003 | 0.025 | 0.003 | 27.90 | 37.30 | 0.10 | 0.50 | 0.50 | 2.00 | 0.10 | 1.10 |
|  | 4 | Ghazvini | 0.00 | 1815.00 | 0.00 | 110.00 | 92.50 | 42.50 | 0.00 | 0.00 | 31.22 | 12.04 | 8.68 | 1.16 | 0.035 | 0.350 | 0.035 | 102.30 | 136.77 | 0.10 | 0.50 | 0.50 | 2.00 | 0.10 | 1.10 |
|  | 5 | Ghazvini | 0.00 | 1815.00 | 0.00 | 1320.00 | 1110.00 | 510.00 | 0.00 | 0.00 | 89.20 | 34.40 | 24.80 | 3.32 | 0.100 | 1.000 | 0.100 | 27.90 | 37.30 | 0.10 | 0.50 | 0.50 | 2.00 | 0.10 | 1.10 |
|  | 6 | Ghazvini | 950.00 | 330.00 | 0.00 | 1320.00 | 1110.00 | 510.00 | 0.00 | 0.00 | 2.23 | 0.86 | 0.62 | 0.08 | 0.003 | 0.025 | 0.003 | 139.50 | 186.50 | 0.10 | 0.50 | 0.50 | 2.00 | 0.10 | 1.10 |
|  | 7 | Ghazvini | 0.00 | 330.00 | 0.00 | 110.00 | 92.50 | 42.50 | 0.00 | 0.00 | 2.23 | 0.86 | 0.62 | 0.08 | 0.003 | 0.025 | 0.003 | 27.90 | 37.30 | 0.10 | 0.50 | 0.50 | 2.00 | 0.10 | 1.10 |
|  | 8 | Ghazvini | 0.00 | 1815.00 | 0.00 | 1320.00 | 1110.00 | 510.00 | 0.00 | 0.00 | 89.20 | 34.40 | 24.80 | 3.32 | 0.100 | 1.000 | 0.100 | 139.50 | 186.50 | 0.10 | 0.50 | 0.50 | 2.00 | 0.10 | 1.10 |
|  | 9 | Ghazvini | 633.33 | 825.00 | 0.00 | 110.00 | 92.50 | 42.50 | 0.00 | 0.00 | 2.23 | 0.86 | 0.62 | 0.08 | 0.003 | 0.025 | 0.003 | 139.50 | 186.50 | 0.10 | 0.50 | 0.50 | 2.00 | 0.10 | 1.10 |
|  | 10 | Ghazvini | 0.00 | 825.00 | 0.00 | 1320.00 | 1110.00 | 510.00 | 0.00 | 0.00 | 2.23 | 0.86 | 0.62 | 0.08 | 0.003 | 0.025 | 0.003 | 65.10 | 87.03 | 0.10 | 0.50 | 0.50 | 2.00 | 0.10 | 1.10 |
|  | 11 | Ghazvini | 1900.00 | 1815.00 | 0.00 | 110.00 | 92.50 | 42.50 | 0.00 | 0.00 | 89.20 | 34.40 | 24.80 | 3.32 | 0.100 | 1.000 | 0.100 | 27.90 | 37.30 | 0.10 | 0.50 | 0.50 | 2.00 | 0.10 | 1.10 |
|  | 12 | Ghazvini | 0.00 | 330.00 | 0.00 | 513.33 | 431.67 | 198.33 | 0.00 | 0.00 | 60.21 | 23.22 | 16.74 | 2.24 | 0.068 | 0.675 | 0.068 | 139.50 | 186.50 | 0.10 | 0.50 | 0.50 | 2.00 | 0.10 | 1.10 |
|  | 13 | Ghazvini | 1900.00 | 1072.50 | 0.00 | 110.00 | 92.50 | 42.50 | 0.00 | 0.00 | 2.23 | 0.86 | 0.62 | 0.08 | 0.003 | 0.025 | 0.003 | 27.90 | 37.30 | 0.10 | 0.50 | 0.50 | 2.00 | 0.10 | 1.10 |
|  | 14 | Ghazvini | 633.33 | 330.00 | 0.00 | 1320.00 | 1110.00 | 510.00 | 0.00 | 0.00 | 89.20 | 34.40 | 24.80 | 3.32 | 0.100 | 1.000 | 0.100 | 102.30 | 136.77 | 0.10 | 0.50 | 0.50 | 2.00 | 0.10 | 1.10 |
|  | 15 | Ghazvini | 1900.00 | 330.00 | 0.00 | 513.33 | 431.67 | 198.33 | 0.00 | 0.00 | 2.23 | 0.86 | 0.62 | 0.08 | 0.003 | 0.025 | 0.003 | 102.30 | 136.77 | 0.10 | 0.50 | 0.50 | 2.00 | 0.10 | 1.10 |
|  | 16 | Ghazvini | 1266.67 | 1815.00 | 0.00 | 1320.00 | 1110.00 | 510.00 | 0.00 | 0.00 | 2.23 | 0.86 | 0.62 | 0.08 | 0.003 | 0.025 | 0.003 | 102.30 | 136.77 | 0.10 | 0.50 | 0.50 | 2.00 | 0.10 | 1.10 |
|  | 17 | Ghazvini | 0.00 | 1815.00 | 0.00 | 715.00 | 601.25 | 276.25 | 0.00 | 0.00 | 2.23 | 0.86 | 0.62 | 0.08 | 0.003 | 0.025 | 0.003 | 27.90 | 37.30 | 0.10 | 0.50 | 0.50 | 2.00 | 0.10 | 1.10 |
|  | 18 | Ghazvini | 1900.00 | 1320.00 | 0.00 | 1320.00 | 1110.00 | 510.00 | 0.00 | 0.00 | 89.20 | 34.40 | 24.80 | 3.32 | 0.100 | 1.000 | 0.100 | 65.10 | 87.03 | 0.10 | 0.50 | 0.50 | 2.00 | 0.10 | 1.10 |
|  | 19 | Ghazvini | 1900.00 | 1320.00 | 0.00 | 110.00 | 92.50 | 42.50 | 0.00 | 0.00 | 60.21 | 23.22 | 16.74 | 2.24 | 0.068 | 0.675 | 0.068 | 139.50 | 186.50 | 0.10 | 0.50 | 0.50 | 2.00 | 0.10 | 1.10 |
|  | 20 | Ghazvini | 1900.00 | 1815.00 | 0.00 | 110.00 | 92.50 | 42.50 | 0.00 | 0.00 | 2.23 | 0.86 | 0.62 | 0.08 | 0.003 | 0.025 | 0.003 | 102.30 | 136.77 | 0.10 | 0.50 | 0.50 | 2.00 | 0.10 | 1.10 |
|  | 21 | Ghazvini | 950.00 | 1072.50 | 0.00 | 715.00 | 601.25 | 276.25 | 0.00 | 0.00 | 45.72 | 17.63 | 12.71 | 1.70 | 0.051 | 0.513 | 0.051 | 83.70 | 111.90 | 0.10 | 0.50 | 0.50 | 2.00 | 0.10 | 1.10 |
|  | 22 | Ghazvini | 1900.00 | 1815.00 | 0.00 | 1320.00 | 1110.00 | 510.00 | 0.00 | 0.00 | 60.21 | 23.22 | 16.74 | 2.24 | 0.068 | 0.675 | 0.068 | 139.50 | 186.50 | 0.10 | 0.50 | 0.50 | 2.00 | 0.10 | 1.10 |
|  | 23 | Ghazvini | 1900.00 | 330.00 | 0.00 | 1320.00 | 1110.00 | 510.00 | 0.00 | 0.00 | 60.21 | 23.22 | 16.74 | 2.24 | 0.068 | 0.675 | 0.068 | 139.50 | 186.50 | 0.10 | 0.50 | 0.50 | 2.00 | 0.10 | 1.10 |
|  | 24 | Ghazvini | 950.00 | 1072.50 | 0.00 | 715.00 | 601.25 | 276.25 | 0.00 | 0.00 | 45.72 | 17.63 | 12.71 | 1.70 | 0.051 | 0.513 | 0.051 | 55.80 | 74.60 | 0.10 | 0.50 | 0.50 | 2.00 | 0.10 | 1.10 |
|  | 25 | Ghazvini | 1266.67 | 330.00 | 0.00 | 1320.00 | 1110.00 | 510.00 | 0.00 | 0.00 | 31.22 | 12.04 | 8.68 | 1.16 | 0.035 | 0.350 | 0.035 | 27.90 | 37.30 | 0.10 | 0.50 | 0.50 | 2.00 | 0.10 | 1.10 |
|  | 26 | Ghazvini | 0.00 | 1320.00 | 0.00 | 1320.00 | 1110.00 | 510.00 | 0.00 | 0.00 | 31.22 | 12.04 | 8.68 | 1.16 | 0.035 | 0.350 | 0.035 | 139.50 | 186.50 | 0.10 | 0.50 | 0.50 | 2.00 | 0.10 | 1.10 |
|  | 27 | Ghazvini | 0.00 | 1320.00 | 0.00 | 110.00 | 92.50 | 42.50 | 0.00 | 0.00 | 89.20 | 34.40 | 24.80 | 3.32 | 0.100 | 1.000 | 0.100 | 65.10 | 87.03 | 0.10 | 0.50 | 0.50 | 2.00 | 0.10 | 1.10 |
|  | 28 | Ghazvini | 633.33 | 1815.00 | 0.00 | 513.33 | 431.67 | 198.33 | 0.00 | 0.00 | 89.20 | 34.40 | 24.80 | 3.32 | 0.100 | 1.000 | 0.100 | 139.50 | 186.50 | 0.10 | 0.50 | 0.50 | 2.00 | 0.10 | 1.10 |
| **Control** | **POM** | **UCB1** | **558.00** | **1532.00** | **555.00** | **35.00** | **468.00** | **223.00** | **321.00** | **24.00** | **41.49** | **10.20** | **4.80** | **0.80** | **0.110** | **0.300** | **0.020** | **31.20** | **41.70** | **5.30** | **0.70** | **0.60** | **0.25** | **0.09** | **1.50** |
|  | **POM** | **Ghazvini** | **558.00** | **1532.00** | **555.00** | **35.00** | **468.00** | **223.00** | **321.00** | **24.00** | **41.49** | **10.20** | **4.80** | **0.80** | **0.110** | **0.300** | **0.020** | **31.20** | **41.70** | **5.30** | **0.70** | **0.60** | **0.25** | **0.09** | **1.50** |
|  | 1 | UCB1 | 837.00 | 2298.00 | 763.13 | 48.13 | 643.50 | 306.63 | 441.38 | 33.00 | 113.13 | 27.81 | 13.09 | 2.18 | 0.300 | 0.818 | 0.055 | 25.35 | 33.88 | 5.30 | 0.70 | 0.60 | 0.25 | 0.09 | 1.50 |
|  | 2 | UCB1 | 725.26 | 766.00 | 832.50 | 52.50 | 702.00 | 334.50 | 481.50 | 36.00 | 20.75 | 5.10 | 2.40 | 0.40 | 0.055 | 0.150 | 0.010 | 93.60 | 125.10 | 5.30 | 0.70 | 0.60 | 0.25 | 0.09 | 1.50 |
|  | 3 | UCB1 | 279.00 | 2298.00 | 277.50 | 17.50 | 234.00 | 111.50 | 160.50 | 12.00 | 20.75 | 5.10 | 2.40 | 0.40 | 0.055 | 0.150 | 0.010 | 15.60 | 20.85 | 5.30 | 0.70 | 0.60 | 0.25 | 0.09 | 1.50 |
|  | 4 | UCB1 | 837.00 | 1446.06 | 277.50 | 17.50 | 234.00 | 111.50 | 160.50 | 12.00 | 124.47 | 30.60 | 14.40 | 2.40 | 0.330 | 0.900 | 0.060 | 93.60 | 125.10 | 5.30 | 0.70 | 0.60 | 0.25 | 0.09 | 1.50 |
|  | 5 | UCB1 | 345.37 | 2106.50 | 277.50 | 17.50 | 234.00 | 111.50 | 160.50 | 12.00 | 33.71 | 8.29 | 3.90 | 0.65 | 0.089 | 0.244 | 0.016 | 83.85 | 112.07 | 5.30 | 0.70 | 0.60 | 0.25 | 0.09 | 1.50 |
| ×POM Medium | 6 | UCB1 | 279.00 | 766.00 | 517.49 | 32.63 | 436.37 | 207.93 | 299.30 | 22.38 | 124.47 | 30.60 | 14.40 | 2.40 | 0.330 | 0.900 | 0.060 | 46.31 | 61.89 | 5.30 | 0.70 | 0.60 | 0.25 | 0.09 | 1.50 |
|  | 7 | UCB1 | 279.00 | 957.50 | 806.47 | 50.86 | 680.05 | 324.04 | 466.44 | 34.87 | 25.61 | 6.30 | 2.96 | 0.49 | 0.068 | 0.185 | 0.012 | 89.82 | 120.05 | 5.30 | 0.70 | 0.60 | 0.25 | 0.09 | 1.50 |
|  | 8 | UCB1 | 837.00 | 2298.00 | 832.50 | 52.50 | 702.00 | 334.50 | 481.50 | 36.00 | 20.75 | 5.10 | 2.40 | 0.40 | 0.055 | 0.150 | 0.010 | 15.60 | 20.85 | 5.30 | 0.70 | 0.60 | 0.25 | 0.09 | 1.50 |
|  | 9 | UCB1 | 479.72 | 766.00 | 277.50 | 17.50 | 234.00 | 111.50 | 160.50 | 12.00 | 71.23 | 17.51 | 8.24 | 1.37 | 0.189 | 0.515 | 0.034 | 15.60 | 20.85 | 5.30 | 0.70 | 0.60 | 0.25 | 0.09 | 1.50 |
|  | 10 | UCB1 | 279.00 | 2298.00 | 832.50 | 52.50 | 702.00 | 334.50 | 481.50 | 36.00 | 124.47 | 30.60 | 14.40 | 2.40 | 0.330 | 0.900 | 0.060 | 93.60 | 125.10 | 5.30 | 0.70 | 0.60 | 0.25 | 0.09 | 1.50 |
|  | 11 | UCB1 | 418.50 | 1155.44 | 832.50 | 52.50 | 702.00 | 334.50 | 481.50 | 36.00 | 102.45 | 25.19 | 11.85 | 1.98 | 0.272 | 0.741 | 0.049 | 15.60 | 20.85 | 5.30 | 0.70 | 0.60 | 0.25 | 0.09 | 1.50 |
|  | 12 | UCB1 | 279.00 | 766.00 | 277.50 | 17.50 | 234.00 | 111.50 | 160.50 | 12.00 | 20.75 | 5.10 | 2.40 | 0.40 | 0.055 | 0.150 | 0.010 | 93.60 | 125.10 | 5.30 | 0.70 | 0.60 | 0.25 | 0.09 | 1.50 |
|  | 13 | UCB1 | 837.00 | 2298.00 | 277.50 | 17.50 | 234.00 | 111.50 | 160.50 | 12.00 | 20.75 | 5.10 | 2.40 | 0.40 | 0.055 | 0.150 | 0.010 | 43.90 | 58.67 | 5.30 | 0.70 | 0.60 | 0.25 | 0.09 | 1.50 |
|  | 14 | UCB1 | 279.00 | 873.22 | 832.50 | 52.50 | 702.00 | 334.50 | 481.50 | 36.00 | 124.47 | 30.60 | 14.40 | 2.40 | 0.330 | 0.900 | 0.060 | 93.60 | 125.10 | 5.30 | 0.70 | 0.60 | 0.25 | 0.09 | 1.50 |
|  | 15 | UCB1 | 837.00 | 766.00 | 538.43 | 33.96 | 454.03 | 216.34 | 311.42 | 23.28 | 76.50 | 18.81 | 8.85 | 1.48 | 0.203 | 0.553 | 0.037 | 93.60 | 125.10 | 5.30 | 0.70 | 0.60 | 0.25 | 0.09 | 1.50 |
|  | 16 | UCB1 | 837.00 | 766.00 | 832.50 | 52.50 | 702.00 | 334.50 | 481.50 | 36.00 | 124.47 | 30.60 | 14.40 | 2.40 | 0.330 | 0.900 | 0.060 | 15.60 | 20.85 | 5.30 | 0.70 | 0.60 | 0.25 | 0.09 | 1.50 |
|  | 17 | UCB1 | 279.00 | 2298.00 | 277.50 | 17.50 | 234.00 | 111.50 | 160.50 | 12.00 | 124.47 | 30.60 | 14.40 | 2.40 | 0.330 | 0.900 | 0.060 | 93.60 | 125.10 | 5.30 | 0.70 | 0.60 | 0.25 | 0.09 | 1.50 |
|  | 18 | UCB1 | 279.00 | 766.00 | 832.50 | 52.50 | 702.00 | 334.50 | 481.50 | 36.00 | 20.75 | 5.10 | 2.40 | 0.40 | 0.055 | 0.150 | 0.010 | 15.60 | 20.85 | 5.30 | 0.70 | 0.60 | 0.25 | 0.09 | 1.50 |
|  | 19 | UCB1 | 418.50 | 1915.00 | 667.89 | 42.12 | 563.20 | 268.36 | 386.29 | 28.88 | 85.22 | 20.95 | 9.86 | 1.64 | 0.226 | 0.616 | 0.041 | 60.75 | 81.20 | 5.30 | 0.70 | 0.60 | 0.25 | 0.09 | 1.50 |
|  | 20 | UCB1 | 653.69 | 766.00 | 277.50 | 17.50 | 234.00 | 111.50 | 160.50 | 12.00 | 124.47 | 30.60 | 14.40 | 2.40 | 0.330 | 0.900 | 0.060 | 56.22 | 75.14 | 5.30 | 0.70 | 0.60 | 0.25 | 0.09 | 1.50 |
|  | 21 | UCB1 | 767.25 | 2250.13 | 763.13 | 48.13 | 643.50 | 306.63 | 441.38 | 33.00 | 28.85 | 7.09 | 3.34 | 0.56 | 0.076 | 0.209 | 0.014 | 93.60 | 125.10 | 5.30 | 0.70 | 0.60 | 0.25 | 0.09 | 1.50 |
|  | 22 | UCB1 | 279.00 | 1373.71 | 277.50 | 17.50 | 234.00 | 111.50 | 160.50 | 12.00 | 124.47 | 30.60 | 14.40 | 2.40 | 0.330 | 0.900 | 0.060 | 15.60 | 20.85 | 5.30 | 0.70 | 0.60 | 0.25 | 0.09 | 1.50 |
|  | 23 | UCB1 | 470.50 | 885.69 | 521.73 | 32.90 | 439.95 | 209.63 | 301.76 | 22.56 | 56.48 | 13.88 | 6.53 | 1.09 | 0.150 | 0.408 | 0.027 | 63.22 | 84.49 | 5.30 | 0.70 | 0.60 | 0.25 | 0.09 | 1.50 |
|  | 24 | UCB1 | 837.00 | 766.00 | 277.50 | 17.50 | 234.00 | 111.50 | 160.50 | 12.00 | 20.75 | 5.10 | 2.40 | 0.40 | 0.055 | 0.150 | 0.010 | 15.60 | 20.85 | 5.30 | 0.70 | 0.60 | 0.25 | 0.09 | 1.50 |
|  | 25 | UCB1 | 348.75 | 2106.50 | 763.13 | 48.13 | 643.50 | 306.63 | 441.38 | 33.00 | 20.75 | 5.10 | 2.40 | 0.40 | 0.055 | 0.150 | 0.010 | 15.60 | 20.85 | 5.30 | 0.70 | 0.60 | 0.25 | 0.09 | 1.50 |
|  | 26 | UCB1 | 665.40 | 1846.56 | 515.51 | 32.51 | 434.70 | 207.13 | 298.16 | 22.29 | 46.68 | 11.48 | 5.40 | 0.90 | 0.124 | 0.338 | 0.023 | 15.60 | 20.85 | 5.30 | 0.70 | 0.60 | 0.25 | 0.09 | 1.50 |
|  | 27 | UCB1 | 837.00 | 2298.00 | 277.50 | 17.50 | 234.00 | 111.50 | 160.50 | 12.00 | 124.47 | 30.60 | 14.40 | 2.40 | 0.330 | 0.900 | 0.060 | 15.60 | 20.85 | 5.30 | 0.70 | 0.60 | 0.25 | 0.09 | 1.50 |
|  | 28 | UCB1 | 279.00 | 2298.00 | 832.50 | 52.50 | 702.00 | 334.50 | 481.50 | 36.00 | 124.47 | 30.60 | 14.40 | 2.40 | 0.330 | 0.900 | 0.060 | 15.60 | 20.85 | 5.30 | 0.70 | 0.60 | 0.25 | 0.09 | 1.50 |
|  | 29 | UCB1 | 837.00 | 2298.00 | 832.50 | 52.50 | 702.00 | 334.50 | 481.50 | 36.00 | 124.47 | 30.60 | 14.40 | 2.40 | 0.330 | 0.900 | 0.060 | 93.60 | 125.10 | 5.30 | 0.70 | 0.60 | 0.25 | 0.09 | 1.50 |
|  | 30 | UCB1 | 837.00 | 1298.97 | 832.50 | 52.50 | 702.00 | 334.50 | 481.50 | 36.00 | 65.55 | 16.11 | 7.58 | 1.26 | 0.174 | 0.474 | 0.032 | 54.77 | 73.20 | 5.30 | 0.70 | 0.60 | 0.25 | 0.09 | 1.50 |
|  | 31 | UCB1 | 279.00 | 2298.00 | 832.50 | 52.50 | 702.00 | 334.50 | 481.50 | 36.00 | 20.75 | 5.10 | 2.40 | 0.40 | 0.055 | 0.150 | 0.010 | 93.60 | 125.10 | 5.30 | 0.70 | 0.60 | 0.25 | 0.09 | 1.50 |
|  | 32 | UCB1 | 279.00 | 766.00 | 277.50 | 17.50 | 234.00 | 111.50 | 160.50 | 12.00 | 124.47 | 30.60 | 14.40 | 2.40 | 0.330 | 0.900 | 0.060 | 93.60 | 125.10 | 5.30 | 0.70 | 0.60 | 0.25 | 0.09 | 1.50 |
|  | 33 | UCB1 | 837.00 | 2298.00 | 277.50 | 17.50 | 234.00 | 111.50 | 160.50 | 12.00 | 20.75 | 5.10 | 2.40 | 0.40 | 0.055 | 0.150 | 0.010 | 93.60 | 125.10 | 5.30 | 0.70 | 0.60 | 0.25 | 0.09 | 1.50 |
|  | 1 | Ghazvini | 837.00 | 2298.00 | 763.13 | 48.13 | 643.50 | 306.63 | 441.38 | 33.00 | 113.13 | 27.81 | 13.09 | 2.18 | 0.300 | 0.818 | 0.055 | 25.35 | 33.88 | 5.30 | 0.70 | 0.60 | 0.25 | 0.09 | 1.50 |
|  | 2 | Ghazvini | 725.26 | 766.00 | 832.50 | 52.50 | 702.00 | 334.50 | 481.50 | 36.00 | 20.75 | 5.10 | 2.40 | 0.40 | 0.055 | 0.150 | 0.010 | 93.60 | 125.10 | 5.30 | 0.70 | 0.60 | 0.25 | 0.09 | 1.50 |
|  | 3 | Ghazvini | 279.00 | 2298.00 | 277.50 | 17.50 | 234.00 | 111.50 | 160.50 | 12.00 | 20.75 | 5.10 | 2.40 | 0.40 | 0.055 | 0.150 | 0.010 | 15.60 | 20.85 | 5.30 | 0.70 | 0.60 | 0.25 | 0.09 | 1.50 |
|  | 4 | Ghazvini | 837.00 | 1446.06 | 277.50 | 17.50 | 234.00 | 111.50 | 160.50 | 12.00 | 124.47 | 30.60 | 14.40 | 2.40 | 0.330 | 0.900 | 0.060 | 93.60 | 125.10 | 5.30 | 0.70 | 0.60 | 0.25 | 0.09 | 1.50 |
|  | 5 | Ghazvini | 345.37 | 2106.50 | 277.50 | 17.50 | 234.00 | 111.50 | 160.50 | 12.00 | 33.71 | 8.29 | 3.90 | 0.65 | 0.089 | 0.244 | 0.016 | 83.85 | 112.07 | 5.30 | 0.70 | 0.60 | 0.25 | 0.09 | 1.50 |
|  | 6 | Ghazvini | 279.00 | 766.00 | 517.49 | 32.63 | 436.37 | 207.93 | 299.30 | 22.38 | 124.47 | 30.60 | 14.40 | 2.40 | 0.330 | 0.900 | 0.060 | 46.31 | 61.89 | 5.30 | 0.70 | 0.60 | 0.25 | 0.09 | 1.50 |
|  | 7 | Ghazvini | 279.00 | 957.50 | 806.47 | 50.86 | 680.05 | 324.04 | 466.44 | 34.87 | 25.61 | 6.30 | 2.96 | 0.49 | 0.068 | 0.185 | 0.012 | 89.82 | 120.05 | 5.30 | 0.70 | 0.60 | 0.25 | 0.09 | 1.50 |
|  | 8 | Ghazvini | 837.00 | 2298.00 | 832.50 | 52.50 | 702.00 | 334.50 | 481.50 | 36.00 | 20.75 | 5.10 | 2.40 | 0.40 | 0.055 | 0.150 | 0.010 | 15.60 | 20.85 | 5.30 | 0.70 | 0.60 | 0.25 | 0.09 | 1.50 |
|  | 9 | Ghazvini | 479.72 | 766.00 | 277.50 | 17.50 | 234.00 | 111.50 | 160.50 | 12.00 | 71.23 | 17.51 | 8.24 | 1.37 | 0.189 | 0.515 | 0.034 | 15.60 | 20.85 | 5.30 | 0.70 | 0.60 | 0.25 | 0.09 | 1.50 |
|  | 10 | Ghazvini | 279.00 | 2298.00 | 832.50 | 52.50 | 702.00 | 334.50 | 481.50 | 36.00 | 124.47 | 30.60 | 14.40 | 2.40 | 0.330 | 0.900 | 0.060 | 93.60 | 125.10 | 5.30 | 0.70 | 0.60 | 0.25 | 0.09 | 1.50 |
|  | 11 | Ghazvini | 418.50 | 1155.44 | 832.50 | 52.50 | 702.00 | 334.50 | 481.50 | 36.00 | 102.45 | 25.19 | 11.85 | 1.98 | 0.272 | 0.741 | 0.049 | 15.60 | 20.85 | 5.30 | 0.70 | 0.60 | 0.25 | 0.09 | 1.50 |
|  | 12 | Ghazvini | 279.00 | 766.00 | 277.50 | 17.50 | 234.00 | 111.50 | 160.50 | 12.00 | 20.75 | 5.10 | 2.40 | 0.40 | 0.055 | 0.150 | 0.010 | 93.60 | 125.10 | 5.30 | 0.70 | 0.60 | 0.25 | 0.09 | 1.50 |
|  | 13 | Ghazvini | 837.00 | 2298.00 | 277.50 | 17.50 | 234.00 | 111.50 | 160.50 | 12.00 | 20.75 | 5.10 | 2.40 | 0.40 | 0.055 | 0.150 | 0.010 | 43.90 | 58.67 | 5.30 | 0.70 | 0.60 | 0.25 | 0.09 | 1.50 |
|  | 14 | Ghazvini | 279.00 | 873.22 | 832.50 | 52.50 | 702.00 | 334.50 | 481.50 | 36.00 | 124.47 | 30.60 | 14.40 | 2.40 | 0.330 | 0.900 | 0.060 | 93.60 | 125.10 | 5.30 | 0.70 | 0.60 | 0.25 | 0.09 | 1.50 |
|  | 15 | Ghazvini | 837.00 | 766.00 | 538.43 | 33.96 | 454.03 | 216.34 | 311.42 | 23.28 | 76.50 | 18.81 | 8.85 | 1.48 | 0.203 | 0.553 | 0.037 | 93.60 | 125.10 | 5.30 | 0.70 | 0.60 | 0.25 | 0.09 | 1.50 |
|  | 16 | Ghazvini | 837.00 | 766.00 | 832.50 | 52.50 | 702.00 | 334.50 | 481.50 | 36.00 | 124.47 | 30.60 | 14.40 | 2.40 | 0.330 | 0.900 | 0.060 | 15.60 | 20.85 | 5.30 | 0.70 | 0.60 | 0.25 | 0.09 | 1.50 |
|  | 17 | Ghazvini | 279.00 | 2298.00 | 277.50 | 17.50 | 234.00 | 111.50 | 160.50 | 12.00 | 124.47 | 30.60 | 14.40 | 2.40 | 0.330 | 0.900 | 0.060 | 93.60 | 125.10 | 5.30 | 0.70 | 0.60 | 0.25 | 0.09 | 1.50 |
|  | 18 | Ghazvini | 279.00 | 766.00 | 832.50 | 52.50 | 702.00 | 334.50 | 481.50 | 36.00 | 20.75 | 5.10 | 2.40 | 0.40 | 0.055 | 0.150 | 0.010 | 15.60 | 20.85 | 5.30 | 0.70 | 0.60 | 0.25 | 0.09 | 1.50 |
|  | 19 | Ghazvini | 418.50 | 1915.00 | 667.89 | 42.12 | 563.20 | 268.36 | 386.29 | 28.88 | 85.22 | 20.95 | 9.86 | 1.64 | 0.226 | 0.616 | 0.041 | 60.75 | 81.20 | 5.30 | 0.70 | 0.60 | 0.25 | 0.09 | 1.50 |
|  | 20 | Ghazvini | 653.69 | 766.00 | 277.50 | 17.50 | 234.00 | 111.50 | 160.50 | 12.00 | 124.47 | 30.60 | 14.40 | 2.40 | 0.330 | 0.900 | 0.060 | 56.22 | 75.14 | 5.30 | 0.70 | 0.60 | 0.25 | 0.09 | 1.50 |
|  | 21 | Ghazvini | 767.25 | 2250.13 | 763.13 | 48.13 | 643.50 | 306.63 | 441.38 | 33.00 | 28.85 | 7.09 | 3.34 | 0.56 | 0.076 | 0.209 | 0.014 | 93.60 | 125.10 | 5.30 | 0.70 | 0.60 | 0.25 | 0.09 | 1.50 |
|  | 22 | Ghazvini | 279.00 | 1373.71 | 277.50 | 17.50 | 234.00 | 111.50 | 160.50 | 12.00 | 124.47 | 30.60 | 14.40 | 2.40 | 0.330 | 0.900 | 0.060 | 15.60 | 20.85 | 5.30 | 0.70 | 0.60 | 0.25 | 0.09 | 1.50 |
|  | 23 | Ghazvini | 470.50 | 885.69 | 521.73 | 32.90 | 439.95 | 209.63 | 301.76 | 22.56 | 56.48 | 13.88 | 6.53 | 1.09 | 0.150 | 0.408 | 0.027 | 63.22 | 84.49 | 5.30 | 0.70 | 0.60 | 0.25 | 0.09 | 1.50 |
|  | 24 | Ghazvini | 837.00 | 766.00 | 277.50 | 17.50 | 234.00 | 111.50 | 160.50 | 12.00 | 20.75 | 5.10 | 2.40 | 0.40 | 0.055 | 0.150 | 0.010 | 15.60 | 20.85 | 5.30 | 0.70 | 0.60 | 0.25 | 0.09 | 1.50 |
|  | 25 | Ghazvini | 348.75 | 2106.50 | 763.13 | 48.13 | 643.50 | 306.63 | 441.38 | 33.00 | 20.75 | 5.10 | 2.40 | 0.40 | 0.055 | 0.150 | 0.010 | 15.60 | 20.85 | 5.30 | 0.70 | 0.60 | 0.25 | 0.09 | 1.50 |
|  | 26 | Ghazvini | 665.40 | 1846.56 | 515.51 | 32.51 | 434.70 | 207.13 | 298.16 | 22.29 | 46.68 | 11.48 | 5.40 | 0.90 | 0.124 | 0.338 | 0.023 | 15.60 | 20.85 | 5.30 | 0.70 | 0.60 | 0.25 | 0.09 | 1.50 |
|  | 27 | Ghazvini | 837.00 | 2298.00 | 277.50 | 17.50 | 234.00 | 111.50 | 160.50 | 12.00 | 124.47 | 30.60 | 14.40 | 2.40 | 0.330 | 0.900 | 0.060 | 15.60 | 20.85 | 5.30 | 0.70 | 0.60 | 0.25 | 0.09 | 1.50 |
|  | 28 | Ghazvini | 279.00 | 2298.00 | 832.50 | 52.50 | 702.00 | 334.50 | 481.50 | 36.00 | 124.47 | 30.60 | 14.40 | 2.40 | 0.330 | 0.900 | 0.060 | 15.60 | 20.85 | 5.30 | 0.70 | 0.60 | 0.25 | 0.09 | 1.50 |
|  | 29 | Ghazvini | 837.00 | 2298.00 | 832.50 | 52.50 | 702.00 | 334.50 | 481.50 | 36.00 | 124.47 | 30.60 | 14.40 | 2.40 | 0.330 | 0.900 | 0.060 | 93.60 | 125.10 | 5.30 | 0.70 | 0.60 | 0.25 | 0.09 | 1.50 |
|  | 30 | Ghazvini | 837.00 | 1298.97 | 832.50 | 52.50 | 702.00 | 334.50 | 481.50 | 36.00 | 65.55 | 16.11 | 7.58 | 1.26 | 0.174 | 0.474 | 0.032 | 54.77 | 73.20 | 5.30 | 0.70 | 0.60 | 0.25 | 0.09 | 1.50 |
|  | 31 | Ghazvini | 279.00 | 2298.00 | 832.50 | 52.50 | 702.00 | 334.50 | 481.50 | 36.00 | 20.75 | 5.10 | 2.40 | 0.40 | 0.055 | 0.150 | 0.010 | 93.60 | 125.10 | 5.30 | 0.70 | 0.60 | 0.25 | 0.09 | 1.50 |
|  | 32 | Ghazvini | 279.00 | 766.00 | 277.50 | 17.50 | 234.00 | 111.50 | 160.50 | 12.00 | 124.47 | 30.60 | 14.40 | 2.40 | 0.330 | 0.900 | 0.060 | 93.60 | 125.10 | 5.30 | 0.70 | 0.60 | 0.25 | 0.09 | 1.50 |
|  | 33 | Ghazvini | 837.00 | 2298.00 | 277.50 | 17.50 | 234.00 | 111.50 | 160.50 | 12.00 | 20.75 | 5.10 | 2.40 | 0.40 | 0.055 | 0.150 | 0.010 | 93.60 | 125.10 | 5.30 | 0.70 | 0.60 | 0.25 | 0.09 | 1.50 |
|  | Min. |  | 0.00 | 330.00 | 0.00 | 17.50 | 92.50 | 42.50 | 0.00 | 0.00 | 2.23 | 0.86 | 0.62 | 0.00 | 0.00 | 0.03 | 0.00 | 15.60 | 20.85 | 0.10 | 0.50 | 0.00 | 0.25 | 0.09 | 1.10 |
|  | Max. |  | 1900.00 | 2298.00 | 1960.00 | 1320.00 | 1110.00 | 510.00 | 1559.00 | 36.00 | 124.47 | 34.40 | 24.80 | 3.32 | 0.33 | 1.00 | 0.10 | 139.50 | 186.50 | 5.30 | 1.00 | 0.60 | 2.00 | 0.10 | 1.50 |

**FIGURE S1|** Graphical representation of the fuzzyfication process developed by neurofuzzy logic per each parameter (A) STN, (B) LN, (C) LC, and (D) BC and their domains (Low, Mid, High) according to^51^.


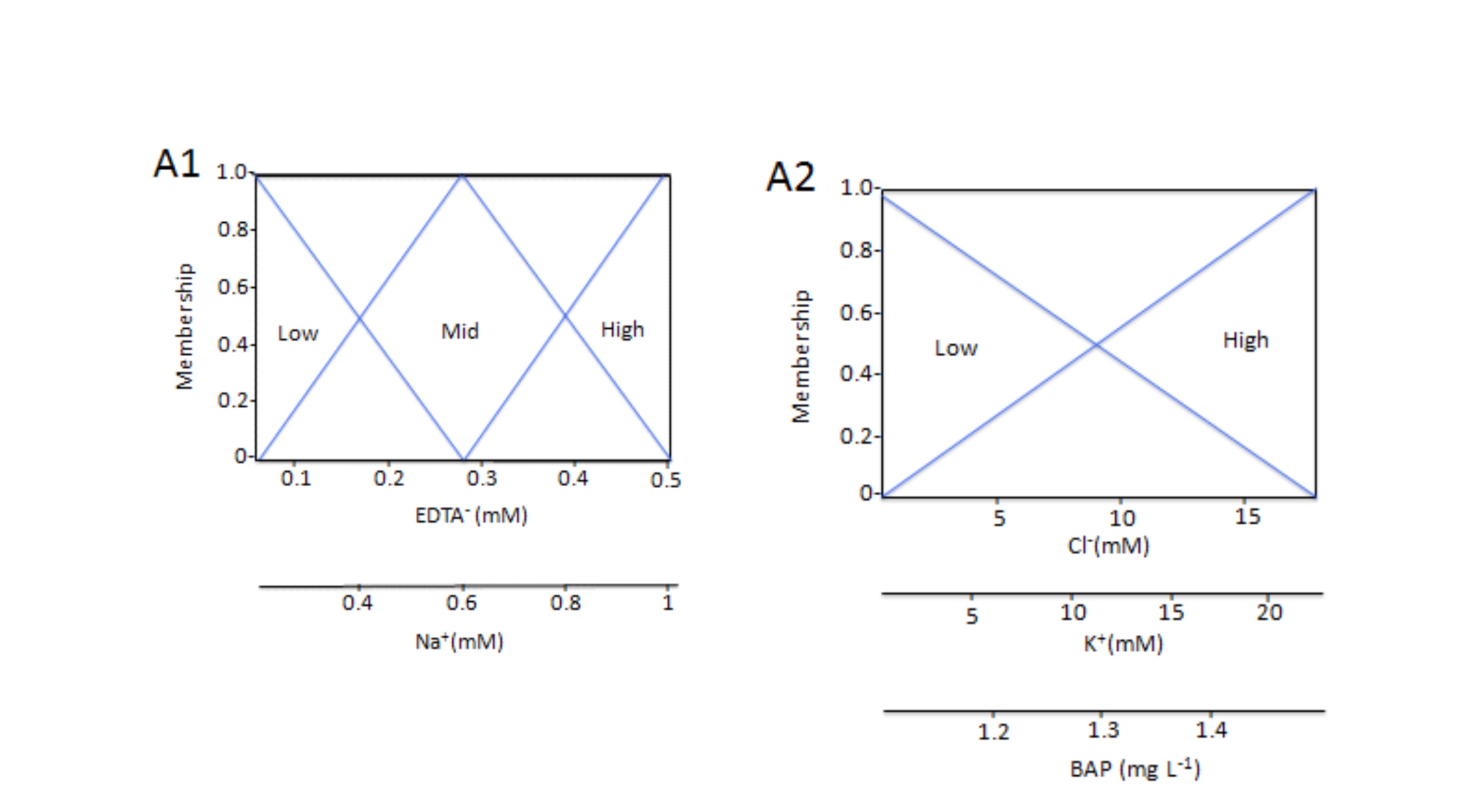


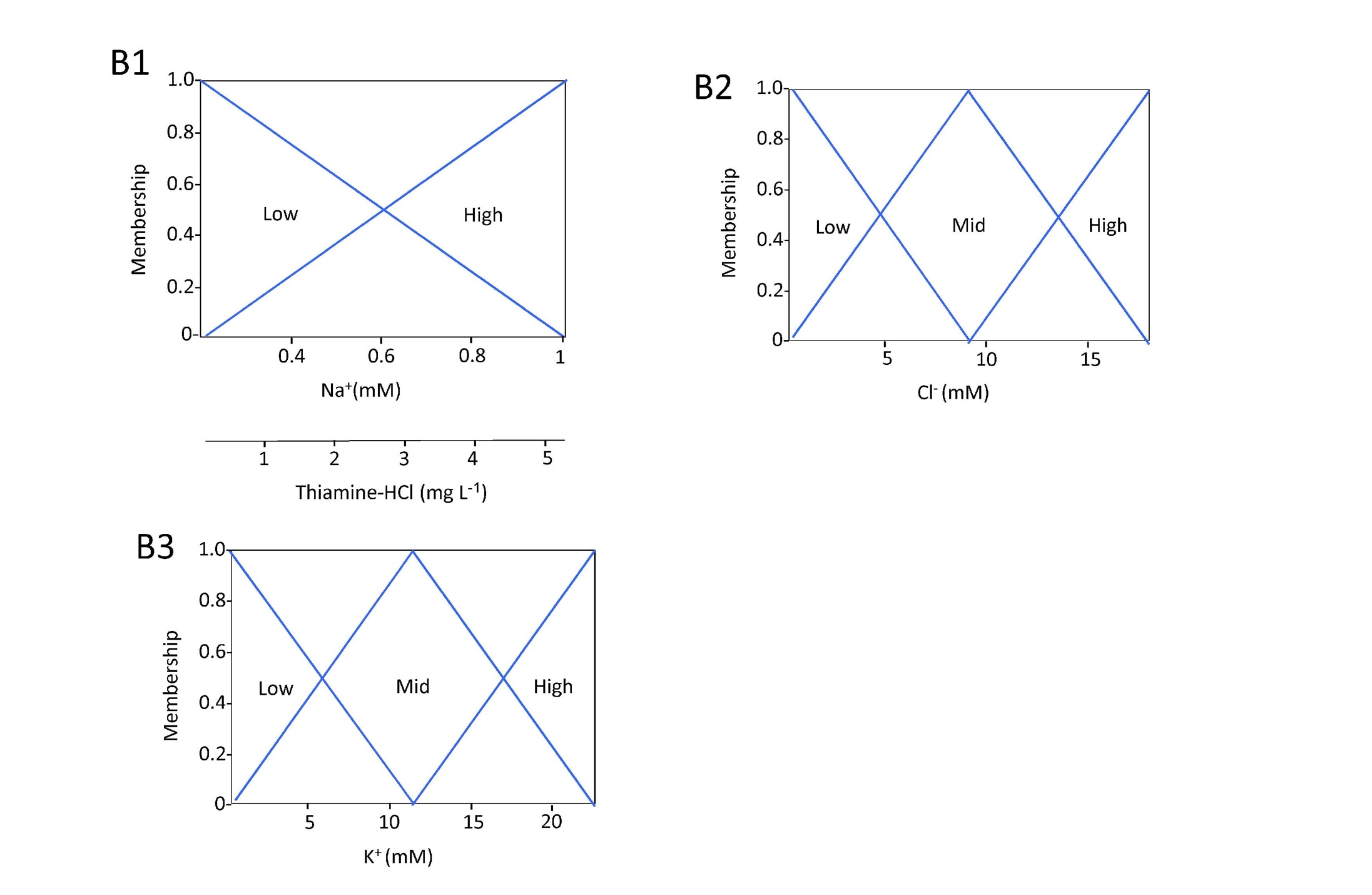


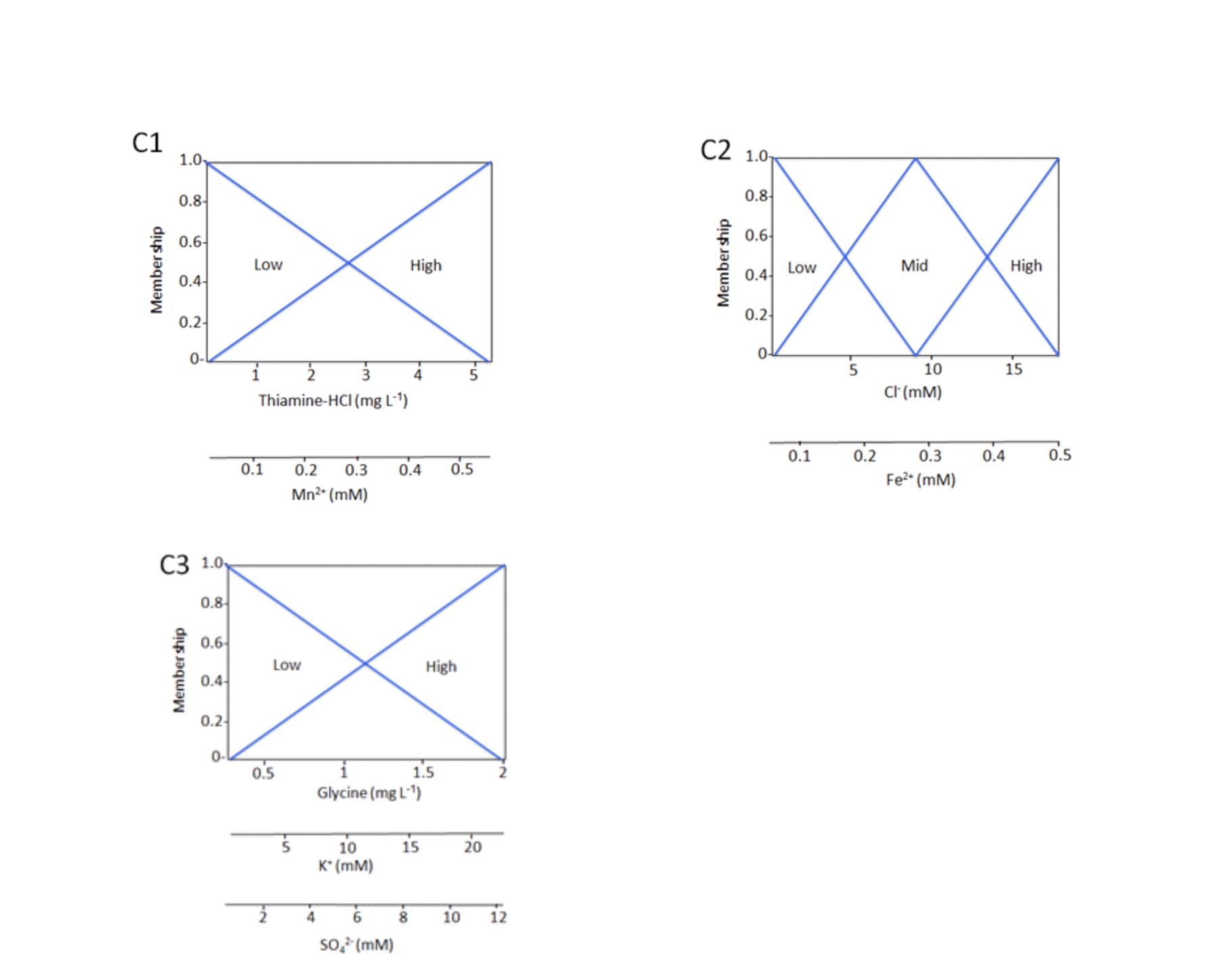


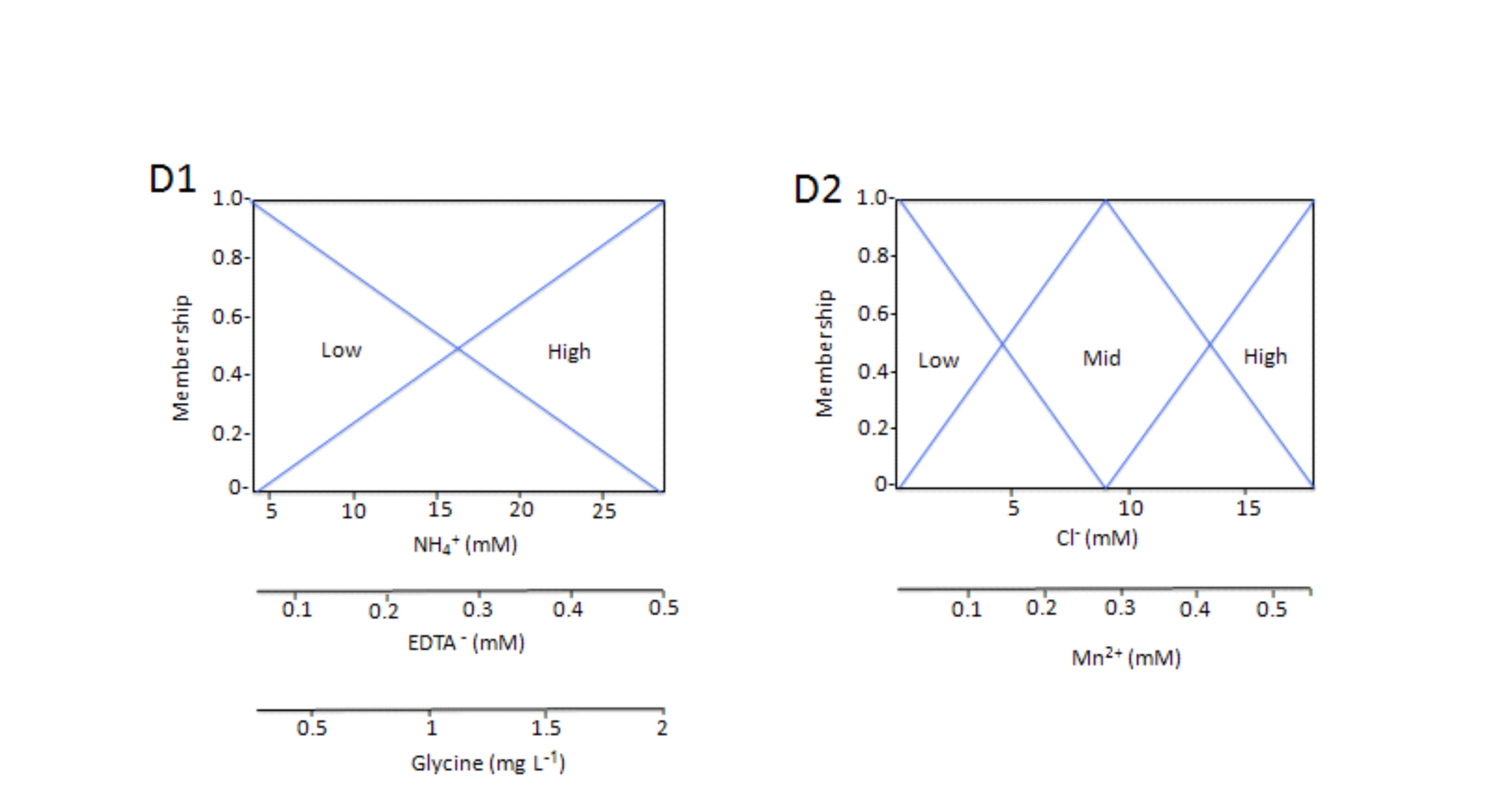

Supplement: Supplementary file 1 — Supplementary Tables and Figure [file 41598_2019_46155_MOESM1_ESM.docx]
